# VIBRANT: automated recovery, annotation and curation of microbial viruses, and evaluation of viral community function from genomic sequences

**DOI:** 10.1186/s40168-020-00867-0

**Published:** 2020-06-10

**Authors:** Kristopher Kieft, Zhichao Zhou, Karthik Anantharaman

**Affiliations:** Department of Bacteriology, University of Wisconsin–Madison, Madison, WI 53706 USA

**Keywords:** Virome, Virus, Bacteriophage, Metagenome, Machine learning, Auxiliary metabolism, Software

## Abstract

**Background:**

Viruses are central to microbial community structure in all environments. The ability to generate large metagenomic assemblies of mixed microbial and viral sequences provides the opportunity to tease apart complex microbiome dynamics, but these analyses are currently limited by the tools available for analyses of viral genomes and assessing their metabolic impacts on microbiomes.

**Design:**

Here we present VIBRANT, the first method to utilize a hybrid machine learning and protein similarity approach that is not reliant on sequence features for automated recovery and annotation of viruses, determination of genome quality and completeness, and characterization of viral community function from metagenomic assemblies. VIBRANT uses neural networks of protein signatures and a newly developed v-score metric that circumvents traditional boundaries to maximize identification of lytic viral genomes and integrated proviruses, including highly diverse viruses. VIBRANT highlights viral auxiliary metabolic genes and metabolic pathways, thereby serving as a user-friendly platform for evaluating viral community function. VIBRANT was trained and validated on reference virus datasets as well as microbiome and virome data.

**Results:**

VIBRANT showed superior performance in recovering higher quality viruses and concurrently reduced the false identification of non-viral genome fragments in comparison to other virus identification programs, specifically VirSorter, VirFinder, and MARVEL. When applied to 120,834 metagenome-derived viral sequences representing several human and natural environments, VIBRANT recovered an average of 94% of the viruses, whereas VirFinder, VirSorter, and MARVEL achieved less powerful performance, averaging 48%, 87%, and 71%, respectively. Similarly, VIBRANT identified more total viral sequence and proteins when applied to real metagenomes. When compared to PHASTER, Prophage Hunter, and VirSorter for the ability to extract integrated provirus regions from host scaffolds, VIBRANT performed comparably and even identified proviruses that the other programs did not. To demonstrate applications of VIBRANT, we studied viromes associated with Crohn’s disease to show that specific viral groups, namely Enterobacteriales-like viruses, as well as putative dysbiosis associated viral proteins are more abundant compared to healthy individuals, providing a possible viral link to maintenance of diseased states.

**Conclusions:**

The ability to accurately recover viruses and explore viral impacts on microbial community metabolism will greatly advance our understanding of microbiomes, host-microbe interactions, and ecosystem dynamics.

Video Abstract

## Background

Viruses that infect bacteria and archaea are globally abundant and outnumber their hosts in most environments [1–3]. Viruses are obligate intracellular pathogenic genetic elements capable of reprogramming host cellular metabolic states during infection and can cause the lysis of 20–40% of microorganisms in diverse environments every day [4, 5]. Due to their abundance and widespread activity, viruses are key facets in microbial communities as they contribute to cycling of essential nutrients such as carbon, nitrogen, phosphorus, and sulfur [6–10]. In human systems, viruses have been implicated in contributing to dysbiosis that can lead to various diseases, such as inflammatory bowel diseases, or even have a symbiotic role with the immune system [11–13].

Viruses harbor vast potential for diverse genetic content, arrangement, and encoded functions [14–17]. Recognizing their genetic diversity, there has been substantial interest in “mining” these viral sequences for novel anti-microbial drug candidates, enzymes for biotechnological applications, and for bioremediation [18–22]. Recently, it has been appreciated that viruses may directly link biogeochemical cycling of nutrients by specifically driving metabolic processes [23–27]. For example, during infection, viruses can acquire 40–90% of their required nutrients from the surrounding environment by taking over and subsequently directing host metabolism [28–30]. To manipulate host metabolic frameworks, some viruses selectively “steal” metabolic genes from their host. These host-derived genes, collectively termed auxiliary metabolic genes (AMGs), can be actively expressed during infection to provide viruses with fitness advantages [31–34]. Due to the need to study the role of viruses in microbiomes and integrate viruses into models of ecosystem function, it has become of great interest to determine which sequences within whole microbial communities are derived from viruses. These sequences can include free virions, active intracellular infections (which may be the case for as many as 30% of all bacteria at any given time [35]), particle or host-attached virions [36], and host-integrated or episomal viral genomes (i.e., proviruses).

Multiple tools exist for the identification of viruses from mixed metagenomic assemblies. For several years, VirSorter [37], which succeeded tools such as VIROME [38] and Metavir [39], has been the most widely used for its ability to identify viral metagenomic fragments (scaffolds) from large metagenomic assemblies. VirSorter predominantly relies on database searches of predicted proteins, using both reference homology as well as probabilistic similarity, to compile metrics of enrichment of virus-like proteins and simultaneous depletion of other proteins. To do this, it uses a virus-specific curated database as well as Pfam [40] for non-virus annotations, though it does not fully differentiate viral from non-viral Pfam annotations. It also incorporates sequence signatures of viral genomes, such as encoding short genes or having low levels of strand switching between genes. VirSorter is also unique in its ability to use these annotations and sequence metrics to identify and extract integrated provirus regions from host scaffolds.

More recent tools have been developed as alternatives or supplements of VirSorter. VirFinder [41] was the first tool to implement machine learning and be completely independent of annotation databases for predicting viruses, which was a platform later implemented in PPR-Meta [42]. VirFinder was built with the consideration that viruses tend to display distinctive patterns of 8-nucleotide frequencies (otherwise known as 8-mers), which was proposed despite the knowledge that viruses can share remarkably similar nucleotide patterns with their host [43]. These 8-mer patterns are used to quickly classify sequences as short as 500 bp and generate model-derived scores, though it is up to the user to define the score cutoffs. VirFinder was shown to greatly improve the ability to recover viruses compared to VirSorter, but it also demonstrated substantial host and source environment biases in predicting diverse viruses, likely due to reference database-associated biases while training the machine learning model [41]. Moreover, under-recovery of viruses from certain environments was identified [44].

Additional recent tools have been developed that utilize slightly different methods for identifying viruses. MARVEL [45], for example, leverages annotation, sequence signatures, and machine learning to identify viruses from metagenomic bins. MARVEL differs from VirSorter in that it only utilizes a single virus-specific database for annotation. However, MARVEL provides no consideration for integrated proviruses and is only suitable for identifying bacterial dsDNA viruses from the order *Caudovirales* which substantially limits its ability to discover novel viruses. Another recently developed tool, VirMiner [46], is unique in that it functions to use metagenomic reads and associated assembly data to identify viruses and performs best for highly abundant viruses. VirMiner is a web-based server that utilizes a hybrid approach of employing both homology-based searches to a virus-specific database as well as machine learning. VirMiner was found to have improved ability to recover viruses compared to both VirSorter and VirFinder but was concurrently much less accurate.

Thus far, VirSorter remains the most efficient tool for identifying integrated proviruses within metagenomic assemblies. Other tools, predominantly PHASTER [47] and Prophage Hunter [48], are specialized in identifying integrated proviruses from whole genomes rather than scaffolds generated by metagenomic assemblies. Similar to VirSorter, these two provirus predictors rely on reference homology and viral sequence signatures with sliding windows to identify regions of a host genome that belong to a virus. Although they are useful for whole genomes, they lack the capability of identifying scaffolds belonging to lytic (i.e., non-integrated) viruses and perform slower for large datasets. In addition, both PHASTER and Prophage Hunter are exclusively available as web-based servers and offer no stand-alone command line tools.

Here we developed VIBRANT (Virus Identification By iteRative ANnoTation), a tool for automated recovery, annotation, and curation of both free and integrated viruses from metagenomic assemblies and genome sequences. VIBRANT is capable of identifying diverse dsDNA, ssDNA, and RNA viruses infecting both bacteria and archaea, and to our knowledge has no evident environmental biases. VIBRANT uses neural networks of protein annotation signatures from non-reference-based similarity searches with Hidden Markov Models (HMMs) as well as a unique “v-score” metric to maximize identification of diverse and novel viruses. After identifying viruses, VIBRANT implements curation steps to validate predictions. VIBRANT additionally characterizes viral community function by highlighting AMGs and assesses the metabolic pathways present in viral communities. All viral genomes, proteins, annotations, and metabolic profiles are compiled into formats for user-friendly downstream analyses and visualization. When applied to reference viruses, non-reference virus datasets, and various assembled metagenomes, VIBRANT outperformed VirFinder, VirSorter, and MARVEL in the ability to maximize virus recovery and minimize false discovery. When compared to PHASTER, Prophage Hunter, and VirSorter for the ability to extract integrated provirus regions from host scaffolds, VIBRANT performed comparably. VIBRANT was also used to identify differences in metabolic capabilities between viruses originating from various environments. When applied to three separate cohorts of individuals with Crohn’s disease, VIBRANT was able to identify both differentially abundant viral groups compared to healthy controls as well as virally encoded genes putatively influencing a diseased state. VIBRANT is freely available for download at https://github.com/AnantharamanLab/VIBRANT. VIBRANT is also available as a user-friendly, web-based application through the CyVerse Discovery Environment at https://de.cyverse.org/de [49].

## Methods

### Dataset for generation and comparison of metrics

To generate training and testing datasets, sequences representing bacteria, archaea, plasmids, and viruses were downloaded from the National Center for Biotechnology Information (NCBI) RefSeq and Genbank databases (accessed July 2019) (Additional File 1: Table S1). For bacteria/archaea, 181 genomes were chosen by selecting from diverse phylogenetic groups. Likewise, a total of 1452 bacterial plasmids were chosen. For viruses, NCBI taxids associated with viruses that infect bacteria or archaea were used to download reference virus genomes, which were then limited to only sequences above 3 kb. This included viruses with both DNA and RNA genomes, though RNA genomes must first be converted to complementary DNA. Sequences not associated with genomes, such as partial genomic regions, were identified according to sequence headers and removed. This resulted in 15,238 total viral partial and complete genomes. To be consistent between all sequences acquired from NCBI, proteins and genes were predicted using Prodigal (-p meta, v2.6.3) [50]. All sequences were split into non-overlapping, non-redundant fragments between 3 and 15 kb to simulate metagenome-assembled scaffolds. These simulated scaffolds are hereafter called *fragments* and were used throughout training and testing VIBRANT. For RNA virus detection, 33 viral (bacteriophage) genomes from NCBI RefSeq and 37 from Krishnamurthy et al. were used [51], and for archaeal virus detection, all genomes were acquired from NCBI RefSeq. The RNA and archaeal viral genomes were represented in both the training and testing datasets as genomic fragments, and recall evaluation was performed on whole genomes. These were the only datasets in which training and evaluation datasets were semi-redundant. See Supplemental Methods (Additional File 16) for additional datasets and sequences used.

Integrated viruses are common in both bacteria and archaea. To address this for generating a dataset devoid of viruses, PHASTER (accessed July 2019) was used to predict putative integrated viruses in the 181 bacteria/archaea genomes. Using BLASTn [52], any fragments that had significant similarity (at least 95% identity, at least 3 kb coverage, and e-value < 1e−10) to the PHASTER predictions were removed as contaminant virus sequence. The new bacteria/archaea dataset was considered depleted of proviruses but not entirely devoid of contamination. Next, the datasets for bacteria/archaea and plasmids were annotated with KEGG, Pfam, and VOG HMMs (hmmsearch (v3.1), e-value < 1e−5) [53] to further remove contaminant virus sequence (see next section for details of HMMs). Plasmids were included because it was noted that the dataset appeared to contain virus sequences, possibly due to misclassification of episomal proviruses as plasmids. Using manual inspection of the KEGG, Pfam, and VOG annotations, any sequence that clearly belonged to a virus was removed. Manual inspection was guided first by the number of KEGG, Pfam, and VOG annotations and then by the annotations themselves. For example, sequences with more VOG than KEGG or Pfam annotations were inspected and removed if multiple viral hallmark genes were found or if the majority of annotations represented viral-like genes. The final datasets consisted of 400,291 fragments for bacteria/archaea, 14,739 for plasmids, and 111,963 for viruses. Total number of fragments for all datasets used can be found in Additional File 2: Table S2.

### Databases used by VIBRANT

VIBRANT uses HMM profiles from three different databases: Kyoto Encyclopedia of Genes and Genomes (KEGG) KoFam (March 2019 release) [54, 55], Pfam (v32) [40], and Virus Orthologous Groups (VOG) (release 94, vogdb.org). For Pfam, all HMM profiles were used. To increase speed, KEGG and VOG HMM databases were reduced in size to contain only profiles likely to annotate the viruses of interest. For KEGG, this was done by only retaining profiles considered to be relevant to “prokaryotes” as determined by KEGG documentation. For VOG, this was done by only retaining profiles that had at least one significant hit to any of the 15,238 NCBI-acquired viruses using BLASTp. The resulting databases consisted of 10,033 HMM profiles for KEGG; 17,929 for Pfam; and 19,182 for VOG (Additional File 3: Table S3).

### V-score generation

Predicted proteins from reference viral genomes from NCBI and VOG database viral proteins were combined to generate v-scores, which resulted in a total of 633,194 proteins. Redundancy was removed from the viral protein dataset using CD-HIT (v4.6) [56] with an identity cutoff of 95%, which resulted in a total of 240,728 viral proteins. This was the final dataset used to generate v-scores. All KEGG HMM profiles were used to annotate the viral proteins. A v-score for each KEGG HMM profile was determined by the number of significant (e-value < 1e−5) hits by hmmsearch, divided by 100, and a maximum value was set at 10 after division. The same v-score generation was done for Pfam and VOG databases. Any HMM profile with no significant hits to the virus dataset was given a v-score of zero. For KEGG and Pfam databases, any annotation that was given a v-score above zero and contained the keyword “phage” was given a minimum v-score of 1. To highlight viral hallmark genes, any annotation within all three databases with the keyword *portal*, *terminase*, *spike*, *capsid*, *sheath*, *tail*, *coat*, *virion*, *lysin*, *holin*, *base plate*, *lysozyme*, *head*, or *structural* was given a minimum v-score of 1. Non-prokaryotic virus annotations (e.g., *reovirus core-spike protein*) were not considered. Each HMM is assigned a v-score and represents a metric of virus association (i.e., do not take into account virus specificity or association with non-viruses) and are manually tuned to put greater weight on viral hallmark genes (Additional File 4: Table S4). Overall, annotations that are likely non-viral will have a low v-score whereas annotations that are commonly associated with viruses will have a high v-score. Raw HMM table outputs for v-score generation can be found in Additional Files 5, 6, and 7 for KEGG, Pfam, and VOG, respectively (Additional File 5: Table S5, Additional File 6: Table S6, and Additional File 7: Table S7).

### Training and testing VIBRANT

The bacteria/archaea genomic, plasmid, and virus datasets described above were used to train and test the machine learning model. Scikit-Learn (v0.21.3) [57] libraries were used to assess various machine learning strategies to identify the best performing algorithm. Among support vector machines, neural networks, and random forests, we found that neural networks lead to the most accurate and comprehensive identification of viruses. Therefore, Scikit-Learn’s supervised neural network multi-layer perceptron classifier (hereafter called neural network) was used. The portion of VIBRANT’s workflow up until the neural network classifier (i.e., KEGG, Pfam, and VOG annotation) was used to compile the 27 annotation metrics for each scaffold. To account for differences in scaffold sizes, all metrics are normalized (i.e., divided by) to the total number of proteins encoded by the scaffold. The first metric, for total proteins, was normalized to log base 10 of itself. Each metric was weighted equally, though it is worth noting that the removal of several metrics did not significantly impact the accuracy of model’s prediction. The normalized results were randomized, and non-redundant portions of these results were taken for training or testing the neural network. In total, 93,913 fragments were used for training, and 9000 different fragments were used for testing the neural network specifically (Additional File 8: Table S8 and Additional File 9: Table S9).

To test the performance of VIBRANT in its entirety, a new testing dataset was generated consisting of fragments from the neural network testing set as well as additional fragments non-redundant to the previous training dataset (hereafter called comprehensive test dataset). This new comprehensive test dataset was comprised of 256,713 genomic fragments from bacteria/archaea, 29,926 from viruses, and 8968 from plasmids. Each met the minimum protein number requirement of VIBRANT: at least four open reading frames.

### Calculation of evaluation metrics and benchmarking of VIBRANT

For comparison of VIBRANT (v1.2.0) to VirFinder (v1.1), VirSorter (v1.0.3), and MARVEL (v0.2), the comprehensive test dataset was used. Two intervals for VirFinder and VirSorter were used for comparison. For VirSorter, the intervals selected were (1) category 1 and 2 predictions, and (2) category 1 and 2 predictions using the *virome decontamination mode*. Categories 1 and 2 are generally considered trustworthy, but category 3 predictions are more likely to contain false identifications. VirSorter was ran using the “Virome” database. For VirFinder, the intervals were (1) scores greater than or equal to 0.90 (approximately equivalent to a *p* value of 0.013) and (2) scores greater than or equal to 0.75 (approximately equivalent to a *p* value of 0.037). Since MARVEL was built for the identification of viral bins, each scaffold was evaluated separately as a single “bin.” To ensure proper identification by MARVEL and VIBRANT, different versions of Scikit-Learn were used for each (v0.19.1 and v0.21.3, respectively).

Several metrics were used to compare performance of all four programs: recall, precision, accuracy, specificity, Mathews Correlation Coefficient (MCC), and F1 score. When calculating metrics, the larger bacteria/archaea and plasmid dataset was normalized to the size of the smaller viral dataset in order to make accurate calculations. All equations used can be found in Additional File 10: Table S10 and the results of each calculation can be found in Additional File 11: Table S11. Comparison metrics were visualized using R (v3.5.2) package “ggplot2.”

It is worth noting that although VIBRANT was tested using sequences that were not used for training, biases may still be associated with reported metrics due to the reliance of KEGG, Pfam, and VOG HMMs on NCBI databases. That is, NCBI databases in part were used to construct the HMMs and therefore are well suited at annotating NCBI-derived sequences. This same type of bias will be seen in the evaluation of VirSorter and MARVEL, both of which rely on NCBI-reliant databases. Although VirFinder does not use annotation databases, the machine-learning algorithm it employs was trained on NCBI-derived sequences. Similarly, biases with comparisons to VirFinder, VirSorter, and MARVEL will arise when using NCBI databases. Sequences from NCBI were used for training each of the three programs and therefore will likely contain redundancy to VIBRANT’s comprehensive test dataset. This redundancy will cause artificially enhanced performance. To address these biases, we further compared all four programs to non-NCBI datasets (see below).

### AMG identification

KEGG annotations were used to classify potential AMGs (Additional File 12: Table S12). KEGG annotations falling under the “metabolic pathways” category as well as “sulfur relay system” were considered. Manual inspection was used to remove non-AMG annotations, such as *nrdAB* and *thyAX*. Other annotations not considered were associated with direct nucleotide to nucleotide conversions. All AMGs were associated with a KEGG metabolic pathway map.

### Completeness estimation

Scaffold completeness is determined based on four metrics: circularization of scaffold sequence, VOG annotations, total VOG nucleotide replication proteins, and total VOG viral hallmark proteins (Additional File 13: Table S13). In order to be considered a complete genome, a sequence must be identified as likely circular. A kmer-based approach is used to do this. Specifically, the first 20 nucleotides are compared to 20-mer sliding windows within the last 900 bp of the sequence. If a complete match is identified, the sequence is considered a circular template. Scaffolds can also be considered a low-, medium-, or high-quality draft. To benchmark completeness, 2466 NCBI RefSeq viruses identified as *Caudovirales*, limited to 10 kb in length, were used to estimate completeness by stepwise removing 10% viral sequence at a time. VIBRANT was found to identify 2465 of the 2466 viruses. This set of viruses was additionally used to assess the error rate of cutting provirus regions. Viral genome diagrams to depict genome quality and completeness, provirus predictions, and novel virus identification were made using Geneious Prime 2019.0.3.

### Analysis of Crohn’s disease metagenomes

Metagenomic reads from He et al*.* [58] were assembled by Pasolli et al*.* [59] and used for analysis. VIBRANT (-l 5000) was used to predict viruses from 49 metagenomes originating from individuals with Crohn’s disease and 53 from healthy individuals (102 total samples). A total of 14,121 viruses were identified. Viral sequences were dereplicated using Mash [60] and Nucmer [61] to 95% nucleotide identity and 70% sequence coverage. The longest sequence was kept as the representative for a total of 8822 dereplicated viruses. A total of 96 read sets were used (59 Crohn’s disease and 37 healthy), trimmed using Sickle and aligned to the dereplicated viruses using Bowtie2 (-N 1, v2.3.4.1) [62], and the resulting coverages were normalized to total reads. The normalized relative coverage of each virus for all 96 samples were compared using DESeq2 [63] (Additional File 14: Table S14). Viruses that displayed significantly different abundance between Crohn’s disease and control samples were determined by a *p* value cutoff of 0.05. iRep (default parameters) [64] was used to estimate replication activity of two highly abundant Crohn’s-associated viruses. EasyFig (v2.2.2) [65] was used to generate genome alignments of Escherichia phage Lambda (NCBI accession number NC_001416.1) and three Crohn’s-associated viruses. vConTACT2 (v0.9.8) was run using default parameters on the CyVerse Discovery Environment platform. Putative hosts of Crohn’s-associated and healthy-associated were estimated using proximity of vConTACT2 protein clustering and BLASTp identity (NCBI non-redundant protein database, assessed October 2019). Two additional read sets from Gevers et al*.* [66] and Ijaz et al*.* [67] were likewise assembled by Pasolli et al. VIBRANT (-l 5000 -o 10) was used to predict viruses from 43 metagenomes originating from individuals with Crohn’s disease and 21 from healthy individuals (64 total samples). In contrast to the discovery, dataset viral genomes were not dereplicated, and differential abundance was not determined. Instead, viruses from each group were directly clustered using vConTACT2. Abundances of dysbiosis-associated genes in the validation set were normalized to total viruses. Validation of dysbiosis-associated genes’ presence on viral genomes, rather than microbial contamination, was done by identifying viral hallmark genes on the viral scaffold (Additional File 15: Table S15). Protein networks were visualized using Cytoscape (v3.7.2) [68].

## Results

VIBRANT was built to extract and analyze bacterial and archaeal viruses from assembled metagenomic and genome sequences, as well as provide a platform for characterizing metabolic proteins and functions in a comprehensive manner. The concept behind VIBRANT’s mechanism of virus identification stems from the understanding that arduous manual inspection of annotated genomic sequences produces the most dependable results. As such, the primary metrics used to inform validated curation standards and to train VIBRANT’s machine learning based neural network to identify viruses reflects human-guided intuition, though in a high-throughput automated fashion.

### Determination of v-score

We developed a unique “v-score” metric as an approach for providing quantitative information to VIBRANT’s algorithm in order to assess the qualitative nature of annotation information. A v-score is a value assigned to each possible protein annotation that scores its association to known viral genomes (see “Methods” section). V-score differs from the previously used “virus quotient” metric [69, 70] in that it does not take into account the annotation’s relatedness to bacteria or archaea. Not including significant similarity to non-viral genomes in the calculation of v-scores has important implications for this metric’s utility. Foremost is that annotations shared between viruses and their hosts, such as ribonucleotide reductases, will be assigned a v-score reflecting its association to viruses, not necessarily virus-specificity. Many genes are commonly associated with viruses and host organisms but when encoded on viral genomes can be central to virus replication efficiency (e.g., ribonucleotide reductases [71]). Therefore, a metric representing virus-association rather than virus-specificity would be more appropriate in identifying if an unknown scaffold is viral or not. Secondly, this approach takes into account widespread horizontal gene transfer of host genes by viruses as well as the presence of AMGs.

### VIBRANT workflow

VIBRANT utilizes several annotation metrics in order to guide removal of non-viral scaffolds before curation of reliable viral scaffolds. The annotation metrics used are derived from HMM-based probabilistic searches of protein families from the KEGG, Pfam, and VOG databases. VIBRANT is not reliant on reference-based similarity and therefore accounts for the large diversity of viruses on Earth and their respective proteins. Consequently, widespread horizontal gene transfer, rapid mutation, and the vast amount of novel sequences do not hinder VIBRANT’s ability to identify known and novel viruses. VIBRANT does not rely on non-annotation features, such as rates of open reading frame strand switching, because these features were not as well conserved in genomic scaffolds in contrast to whole genomes.

**Fig. 1:**
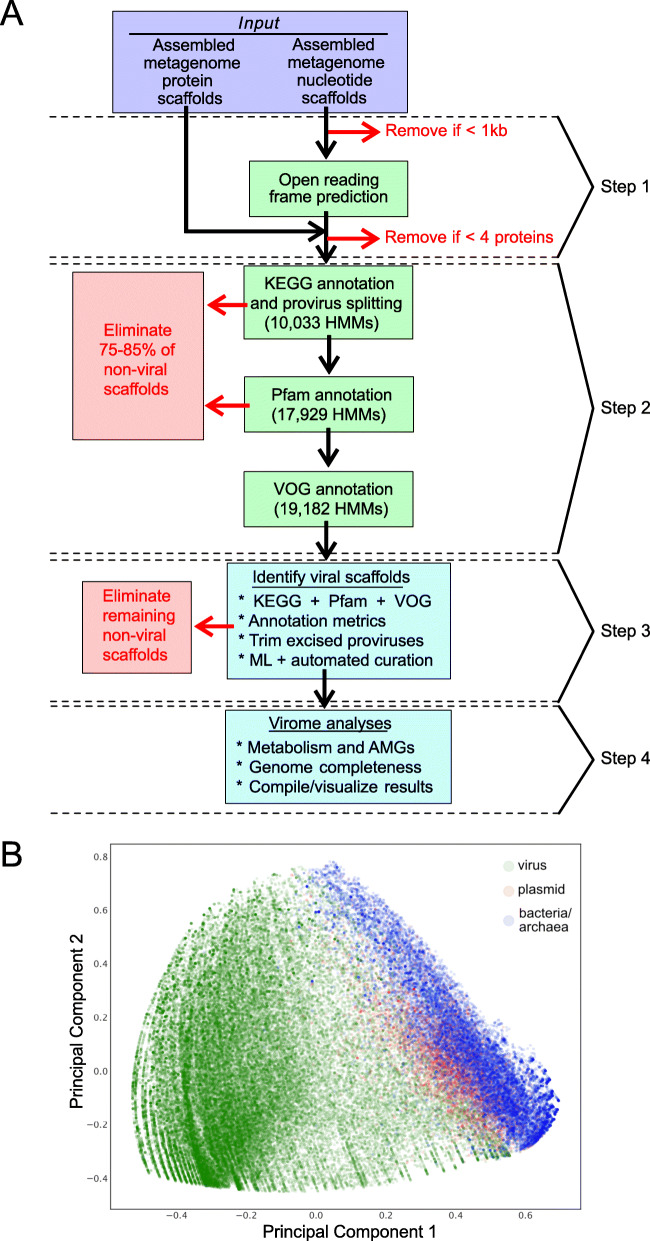
Representation of VIBRANT’s method for virus identification and virome functional characterization. **a** Workflow of virome analysis. Annotations from KEGG, Pfam, and VOG databases are used to construct signatures of viral and non-viral annotation signatures that are read into a neural network machine learning model. **b** Visual representation (PCA plot) of the metrics used by the neural network to identify viruses, depicting viral, plasmid, and bacterial/archaeal genomic sequences

VIBRANT’s workflow consists of four main steps (Fig. 1a). Briefly, proteins (predicted or user input) are used by VIBRANT to first eliminate non-viral sequences by assessing non-viral annotation signatures derived from KEGG and Pfam HMM annotations. At this step, potential host scaffolds are fragmented using sliding windows of KEGG annotation v-scores in order to extract integrated provirus sequences. Following the elimination of most non-viral scaffolds and rough excision of provirus regions, proteins are annotated by VOG HMMs. Before analysis by the neural network machine learning model, any extracted putative provirus is trimmed to exclude any remaining non-viral sequences. Annotations from KEGG, Pfam, and VOG are used to compile 27 metrics that are utilized by the neural network to predict viral sequences (Additional File 16: Supplemental Methods). These 27 metrics were found to be adequate for the separation of viral and non-viral scaffolds (Fig. 1b).

After prediction by the neural network, a set of curation steps are used to filter the results. Curation is an automated mechanism of verifying and/or altering the neural network predictions in order to improve accuracy and recovery of viruses. This concept, as previously stated, originates from experiences with manual inspection of viral genomes that cannot be captured even within machine-learning algorithms. For example, these curation steps can (1) more accurately separate plasmid sequences by discerning viral-like and plasmid-like integrase annotations, (2) remove scaffolds that encode a high density of bacterial-like (i.e., v-score of zero) proteins, or (3) increase true positive identifications by retaining otherwise missed scaffolds that are unique (e.g., encode few but highly virus-related proteins).

Once viruses are identified VIBRANT automates the analysis of viral community function by highlighting AMGs and assigning them to KEGG metabolic pathways. The genome quality (i.e., proxy of completeness) of identified viruses is estimated using a subset of the annotation metrics, and viral sequences are used to identify circular templates (i.e., likely complete circular viruses). These quality analyses were determined to best reflect established completeness metrics for both bacteria and viruses [72, 73]. Finally, VIBRANT compiles all results into a user-friendly format for visualization and downstream analysis. For a detailed description of VIBRANT’s workflow see “Methods” section.

### Comparison of VIBRANT to other programs

VirSorter, VirFinder, and MARVEL, three commonly used programs for identifying bacterial and archaeal viruses from metagenomes, were selected to compare against VIBRANT for the ability to accurately identify viruses. We evaluated all four programs’ performance on the same viral, bacterial, and archaeal genomic and plasmid datasets. Given that both VirSorter and VirFinder produce various confidence ranges of virus identification, we selected certain parameters for each program for comparison. For VirSorter, the parameters selected were (1) category 1 and 2 predictions, and (2) category 1 and 2 predictions using the *virome decontamination mode*. For VirFinder, the intervals were (1) scores greater than or equal to 0.90 (approximately equivalent to a *p* value of 0.013) and (2) scores greater than or equal to 0.75 (approximately equivalent to a *p* value of 0.037). Hereafter, we provide two statistics for each VirSorter and VirFinder run that reflects results according to the two set confidence intervals, respectively. Both VIBRANT and MARVEL have set output predictions and therefore will be reported with a single statistic.

**Fig. 2:**
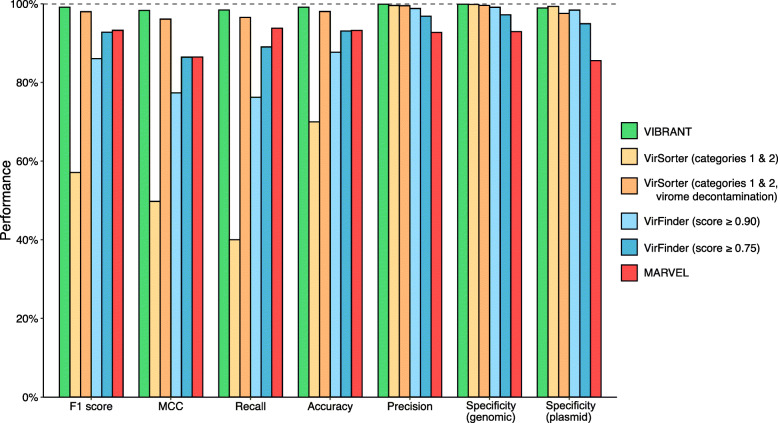
Performance comparison of VIBRANT, VirFinder, VirSorter, and MARVEL on artificial scaffolds of 3–15 kb. Performance was evaluated using datasets of reference viruses, bacterial plasmids, and bacterial/archaeal genomes. For VirFinder and VirSorter, two different confidence cutoffs were used (VirFinder: score of at least 0.90 and score of at least 0.75, VirSorter: categories 1 and 2 predictions and categories 1 and 2 predictions using virome decontamination mode). All four programs were compared using the following statistical metrics: F1 score, MCC, recall, precision, accuracy, and specificity. To ensure equal comparison all scaffolds tested encoded at least four open reading frames

VIBRANT yields a single output of confident predictions and therefore does not provide multiple output options. Since VIBRANT is only partially reliant on its neural network machine learning model for making predictions, all comparisons are focused on VIBRANT’s full workflow performance. VIBRANT does not consider scaffolds shorter than 1000 bp or those that encode less than four predicted open reading frames in order to maintain a low false positive rate (FPR) and have sufficient annotation information for identifying viruses. Therefore, in comparison of performance metrics, only scaffolds meeting VIBRANT’s minimum requirements were analyzed. Inclusion of fragments encoding less than four open reading frames in analyses, which are frequently generated by metagenomic assemblies, is discussed below. We used the following statistics to compare performance: recall, precision, accuracy, specificity, MCC, and F1 score (Fig. 2).

First, we evaluated the true positive rate (TPR or recall) of viral genomic fragments as well as whole viral genomes. Viral genomes were acquired from the NCBI RefSeq and GenBank databases and split into various non-redundant fragments between 3 and 15 kb to simulate genomic scaffolds (see “Methods” section). VIBRANT correctly identified 98.43% of the 29,926 viral fragments, which was greater than VirSorter (40.03% and 96.53%), VirFinder (76.23% and 89.03%), and MARVEL (93.79%) at all scoring intervals. For VirSorter, it was essential to set *virome decontamination mode* for datasets consisting of mainly viruses, without which the TPR was substantially inhibited.

Similar to TPR, we calculated FPR (or specificity) using two different datasets: genomic fragments of bacteria and archaea (hereafter called genomic) and bacterial plasmids (plasmid). Plasmids were evaluated separately because they often encode for genes similar to those on viral genomes, such as those for genome replication and mobilization. Genomic and plasmid sequences were acquired from NCBI RefSeq and GenBank databases and split into various non-redundant fragments between 3 and 15 kb, and putative proviruses were depleted from the datasets (see “Methods” section). VIBRANT had high specificity against both genomic (99.90%) and plasmid fragments (98.90%). VirSorter had similar specificity against both genomic (99.84% and 99.59%) and plasmid (99.33% and 97.55%) datasets, but only VirFinder set to a score cutoff of 0.90 was fully comparable (genomic 99.10%, plasmid 98.39%). VirFinder at a score cutoff of 0.75 (genomic 97.19%, plasmid 94.93%) along with MARVEL (genomic 92.92%, plasmid 85.54%) were slightly less specific. Although VirFinder (set to a score cutoff of 0.90) and VIBRANT had a similar overall specificity, VirFinder identified 9.3 times more genomic scaffolds as viruses (false discoveries) compared to VIBRANT (2311 and 249, respectively). MARVEL was even more pronounced, identifying 72.9 times more genomic scaffolds as viruses (18,164 total) compared to VIBRANT.

We used the results from TPR of viral fragments and FPR of non-viral genomic or plasmid fragments to calculate precision (i.e., proportion of true virus identifications out of all virus identifications) and accuracy (i.e., proportion of correct predictions out of all predictions). VIBRANT outperformed each other program at both precision (VIBRANT 99.87%, VirFinder 98.80% and 96.85%, VirSorter 99.57% and 99.50%, and MARVEL 92.73%) and accuracy (VIBRANT 99.15%, VirFinder 87.67% and 93.08%, VirSorter 69.97% and 98.03%, and MARVEL 93.23%). F1 and MCC are additional metrics (maximum values of 1) accounting for both TPR and FPR, and therefore acts as a comprehensive evaluation of overall performance. Our calculation of F1 indicates that VIBRANT (0.991) is able to better identify viruses while subsequently reducing false identifications compared to VirFinder (0.861 and 0.928), VirSorter (0.571 and 0.980), or MARVEL (0.933). MCC likewise indicated that VIBRANT (0.983) was better suited at maximizing the ratio of viruses to non-viruses compared to VirSorter (0.498 and 0.961), VirFinder (0.774 and 0.864), and MARVEL (0.865).

Although VIBRANT exhibits improved performance with scaffolds at least 3 kb in length, it is worth noting that performance drops considerably at the set minimum length of 1 kb. To display this, the TPR and FPR of both 1 k and 3 kb scaffolds were assessed (Additional File 16: Figure S1A). For this analysis, VirSorter was evaluated using virome decontamination mode and VirFinder was set to a score cutoff of 0.90. MARVEL’s minimum length requirement is 2 kb and therefore was not compared with 1 kb scaffolds. For 1 kb viral scaffolds, VIBRANT (1.95%) and VirSorter (1.12%) recovered far fewer scaffolds compared to VirFinder (22.56%). However, at a length of 3 kb, VIBRANT (43.54%) recovered more viral fragments than VirSorter (25.43%), VirFinder (34.42%), and MARVEL (37.82%). Even at the low resolution of short scaffolds, VIBRANT’s FPR is not impacted. For 1 kb genomic and 1 kb plasmid scaffolds, VIBRANT (< 0.00% and 0.07%) and VirSorter (<0.00% and 0.10%) had fewer false positive discoveries than VirFinder (2.61% and 3.70%). Similarly, for 3 kb genomic and 3 kb plasmid scaffolds, VIBRANT (0.10% and 2.69%) and VirSorter (0.11% and 2.41%) falsely identified fewer sequences than VirFinder (2.26% and 5.54%) or MARVEL (6.08% and 16.30%). Overall, this suggests that VirFinder is uniquely able to accurately recover short (e.g., 1 kb) viral scaffolds while maintaining a relatively low FPR, but this ability is not maintained with longer scaffolds. Moreover, our current abilities to sequence and assemble scaffolds of lengths over 3 kb will likely lead to a greater focus on longer viral sequences that are more amenable to downstream analysis, such as taxonomic classification and functional analyses.

Next, we assessed the ability of VIBRANT to filter out eukaryotic contamination rather than falsely identify these sequences as viral since eukaryotes were not represented in the training or testing datasets. However, these contaminants should be sparse because the majority of eukaryotic KEGG and VOG HMMs were removed from the annotation databases (see “Methods” section). Likewise, eukaryotic-like annotations should receive a low v-score. A total of 8672 eukaryotic sequences ranging from 1 to 15 kb were assessed. VIBRANT (0.62%), VirSorter (0.05% and 0.05%), and MARVEL (0.44%) performed well with recovering few sequences, whereas VirFinder (4.92% and 15.44%) recovered contamination at a greater rate (Additional File 16: Figure S1B).

Finally, viruses with RNA genomes as well as those that infect archaea are rare in current culture systems and sequence databases compared to bacterial dsDNA viruses. However, the true abundance of RNA and archaeal viruses has yet to be explored mainly due to biases towards dsDNA in genome extracting and sequencing methods [74] and the low abundance of archaea in most environments. VIBRANT was built to identify all prokaryotic viruses in order to expand our knowledge of understudied groups. A total of 70 RNA viral genomes and 93 archaeal viral genomes were used to evaluate recall. VIBRANT was able to recover 47% of RNA viruses or 84% of those that encoded at least four predicted open reading frames. In comparison, VirSorter (7% and 70%), VirFinder (33% and 57%), and MARVEL (68%) ranged from lower to higher recovery (Additional File 16: Figure S1C). The high recovery of RNA viruses by MARVEL is intriguing since the software was trained exclusively on dsDNA *Caudovirales* but may be explained by the greater rate of false positive discovery. For archaeal viruses, VIBRANT (96.77%) identified significantly more viruses than VirSorter (70.97% and 93.55%), VirFinder (46.24% and 74.19%), and MARVEL (80.65%) (Additional File 16: Figure S1D). Taken together, VIBRANT has the potential to identify RNA and archaeal viruses, though the significance of this difference is hard to distinguish due to the current dearth of reference genomes with which to validate.

### Identification of viruses in diverse environments

**Fig. 3:**
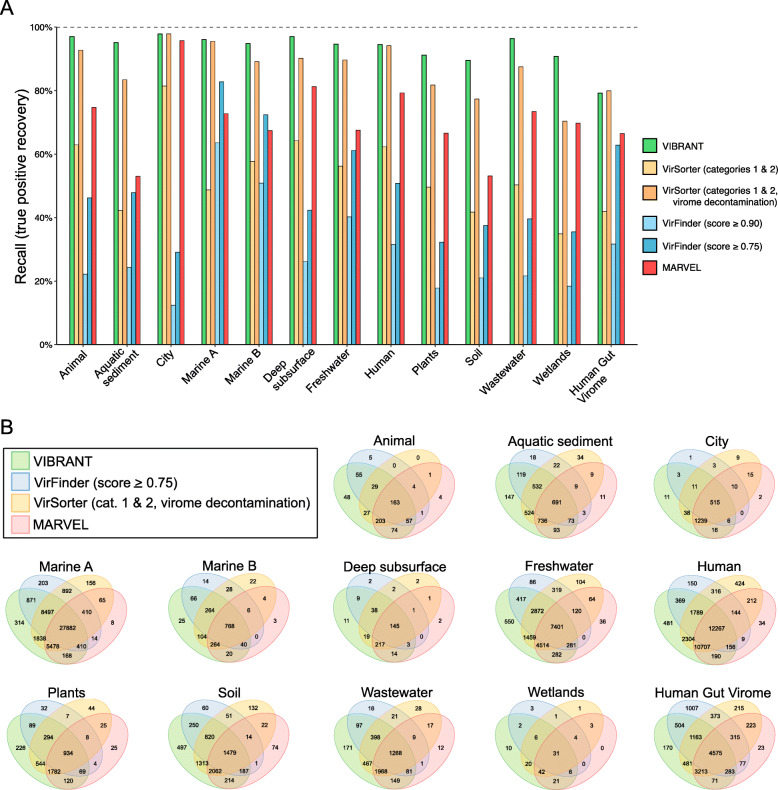
Effect of source environment on predictive abilities of VIBRANT, VirFinder, VirSorter, and MARVEL. Viral scaffolds from IMG/VR and HGV database were used to test if VIBRANT displays biases associated with specific environments. **a** The recall (or recovery) of viral scaffolds from 12 environment groups was compared between VIBRANT and two confidence cutoffs for both VirFinder and VirSorter. Marine environments were classified into two groups: marine A (coastal, gulf, inlet, intertidal, neritic, oceanic, pelagic, and strait) and marine B (hydrothermal vent, volcanic, and oil). **b** Comparison of the overlap in the scaffolds identified as viruses by all three programs. Cutoffs for VirFinder (scores greater than or equal to 0.75) and VirSorter (categories 1 and 2 using virome decontamination mode) were set to display each program’s ability to recover diverse viruses

We next tested VIBRANT’s ability to successfully identify viruses from a diversity of environments. Using 120,834 viruses from the IMG/VR database, in which the source environment of viruses is categorized, we identified that VIBRANT is more robust in identifying viruses from all tested environments compared to VirFinder, VirSorter, and MARVEL (Fig. 3a). The 12 environments were animal-associated, aquatic sediment, city, marine A (coastal, gulf, inlet, intertidal, neritic, oceanic, pelagic, and strait), marine B (hydrothermal vent, volcanic, and oil), deep subsurface, freshwater, human-associated, plant-associated, soil, wastewater, and wetlands. VIBRANT averaged 94.59% recall, substantially greater than VirFinder (29.19% and 48.13%), VirSorter (54.37% and 87.49%), and MARVEL (71.23%). Between the 12 environments, VIBRANT recovered between 89.55% and 97.87% (total range of 8.33%) of the viruses. Conversely, VirFinder (score cutoff of 0.75) had a range of 53.65%, VirSorter (categories 1 and 2, virome decontamination) had a range of 27.48%, and MARVEL had a range of 42.75%. These results suggest that in comparison to other software, VIBRANT has no evident environmental biases and is fully capable of identifying viruses from a broad range of source environments. We also used a dataset of 13,203 viruses from the Human Gut Virome database for additional comparison. The vast majority of viruses (~ 96%) in this dataset were assumed to infect bacteria. Although recall was diminished compared to IMG/VR datasets, VIBRANT (79.22%) nevertheless outperformed or matched VirFinder (31.67% and 62.83%), VirSorter (41.93% and 79.97%), and MARVEL (66.49%) on this dataset.

Relatively few viruses from the IMG/VR dataset that were not identified by VIBRANT were identified by either VirFinder, VirSorter, or MARVEL at even the most inclusive score cutoffs (Fig. 3b). Furthermore, for most environments, VIBRANT displayed the largest proportion of unique identifications, suggesting that VIBRANT has the propensity for discovery of viruses. The differences in the overlap of identified viruses was not too distinctive in environments for which many reference viruses are available, such as marine, though for more understudied environments, such as plants or wastewater, VIBRANT displayed near-complete overlap with VirFinder, VirSorter, and MARVEL predictions. This suggests that database bias may not affect VIBRANT’s performance to a significant degree. Although VirFinder does not rely on an annotation database, it still has been trained on a dataset of reference viral genomes which can contribute to database dependency and recall bias.

### Identification of viruses in mixed metagenomes

Metagenomes assembled using short read technology contain many scaffolds that do not meet VIBRANT’s minimum length requirements and therefore are not considered during analysis. Despite this, VIBRANT’s predictions contain more annotation information and greater total viral sequence length than tools built to identify short sequences, such as scaffolds with less than four open reading frames. VIBRANT, VirFinder (score cutoff of 0.90), and VirSorter (categories 1 and 2) were used to identify viruses from human gut, freshwater lake, and thermophilic compost metagenome sequences (Table 1). In addition, alternate program settings—VIBRANT *virome* mode, VirFinder at a score cutoff of 0.75, and VirSorter virome decontamination mode—were used to identify viruses from an estuary virome dataset. MARVEL was not considered in this analysis due to the inability to achieve comparable precision. Each metagenomic assembly was limited to sequences of at least 1000 bp but no minimum open reading frame limit was set. For these metagenomes, 31 to 40% of the scaffolds were of sufficient length (at least four open reading frames) to be analyzed by VIBRANT; for the estuary virome, 62% was of sufficient length. In comparison, 100% of scaffolds from each dataset was long enough to be analyzed by VirFinder. The ability of VirFinder to make a prediction with each scaffold is considered the major strength of the tool.

**Table 1:** Virus recovery of VIBRANT, VirFinder, and VirSorter from mixed metagenomes and a virome

Metagenome	Seqs. total (≥ 1 kb)	Seqs. ≥ 4 ORFs	Metric	VIBRANT	VirFinder (score ≥ 0.90)	VIBRANT vs. VirFinder	VirSorter(cat. 1 and 2)	VIBRANT vs. VirSorter
Human gut: adenoma	34,883	11,360	Total putative viruses	527	604	0.87	284	**1.86**
			Total virus length (bp)	c5,234,242	1,696,118	**3.09**	3,982,292	**1.31**
			Total virus proteins	7,661	2,134	**3.59**	5,484	**1.40**
Human gut: carcinoma	53,946	18,669	Total putative viruses	784	1,329	0.59	450	**1.74**
			Total virus length (bp)	5,611,953	3,500,838	**1.60**	4,182,862	**1.34**
			Total virus proteins	8,401	4,644	**1.81**	5,945	**1.41**
Human gut: healthy	42,739	17,079	Total putative viruses	565	672	0.84	309	**1.83**
			Total virus length (bp)	5,623,082	2,411,049	**2.33**	4,512,571	**1.25**
			Total virus proteins	8,202	3,230	**2.54**	6,127	**1.34**
Thermophilic compost	68,815	21,620	Total putative viruses	1,047	878	**1.19**	383	**2.73**
			Total virus length (bp)	10,253,162	2,238,129	**4.58**	3,290,654	**3.12**
			Total virus proteins	9,912	2,806	**3.53**	4,400	**2.25**
Freshwater lake (bog)	79,862	26,832	Total putative viruses	5,626	7,567	0.74	1,503	**3.74**
			Total virus length (bp)	34,976,570	25,357,664	**1.38**	15,436,797	**2.27**
			Total virus proteins	56,120	37,537	**1.50**	21,280	**2.64**
*Estuary virome	5,247	3,277	Total putative viruses	3,141	2,294	**1.37**	1,121	**2.80**
			Total virus length (bp)	6,591,285	6,478,804	**1.02**	5,163,674	**1.28**
			Total virus proteins	20,500	12,035	**1.70**	9,645	**2.13**

Mixed community assembled metagenomes from human gut, thermophilic compost, and freshwater, as well as an estuary virome, were used to compare virus prediction ability between the three programs. For each assembly, the scaffolds were limited to a minimum length of 1000 bp. Only a subset of each dataset contained scaffolds encoding at least four open reading frames. VIBRANT, VirFinder (score minimum of 0.90), and VirSorter (categories 1 and 2) were compared by total viral predictions, total combined length of predicted viruses, and total combined proteins of predicted viruses. Comparison columns, denoted “VIBRANT vs. VirFinder” and “VIBRANT vs. VirSorter”, display the comparison ratio of the given metric; bold indicates greater performance by VIBRANT. The asterisk represents that VIBRANT, VirFinder and VirSorter were ran with alternate settings (see Methods)

For all six assemblies, VirFinder averaged approximately 1.16 times more virus identifications than VIBRANT, though for both thermophilic compost and the estuary virome VIBRANT identified a greater number. Despite VirFinder averaging more total virus identifications, VIBRANT averaged 2.33 times more total viral sequence length and 2.44 times more total viral proteins. This is the result of VIBRANT having the capability to identify more viruses of higher quality and longer sequence length. For example, among all six datasets, VIBRANT identified 1320 total viruses at least 10 kb in length in comparison to VirFinder’s 479.

VIBRANT was also able to outperform VirSorter in all metrics, averaging 2.45 times more virus identifications, 1.76 times more total viral sequence length, and 1.86 times more encoded viral proteins.

VIBRANT’s method of predicting viruses provides a unique opportunity in comparison to similar tools in that it yields sequences of higher quality which are more amenable for analyzing protein function from virome data. It is an important distinction that the total number of viruses identified may not be correlated with the total viruses identified or the total number of encoded proteins. Even if VIBRANT identified fewer total viruses compared to other tools in certain circumstances, more data of higher quality was generated as viral sequences of longer length were identified as compared to many short fragments. This provides an important distinction that the metric of total viral predictions is not necessarily an accurate representation for the quality or quantity of the data generated.

### Integrated provirus prediction

**Fig. 4:**
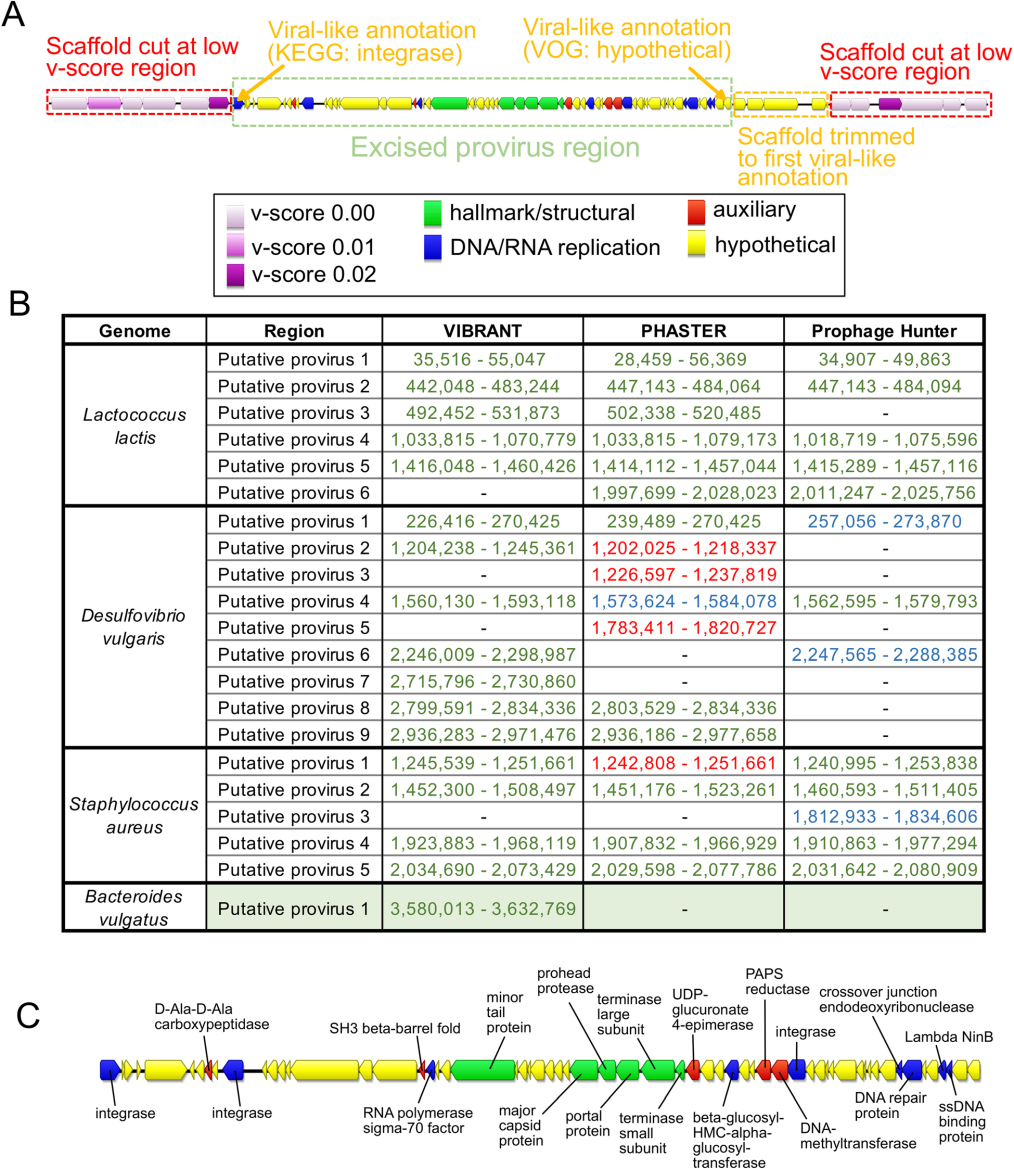
Prediction of integrated proviruses by VIBRANT and comparison to PHASTER, Prophage Hunter, and VirSorter. **a** Schematic representing the method used by VIBRANT to identify and extract provirus regions from host scaffolds using annotations. Briefly, v-scores are used to cut scaffolds at host-specific sites and fragments are trimmed to the nearest viral annotation. **b** Comparison of proviral predictions within four complete bacterial genomes between VIBRANT, PHASTER, Prophage Hunter, and VirSorter. For PHASTER, putative proviruses are colored according to “incomplete” (red), “questionable” (blue), and “intact” (green) predictions. Prophage Hunter is colored according to “active” (green) and “ambiguous” (blue) predictions. All VirSorter predictions for categories 1 and 2 are shown in green. **c** Manual validation of the *Bacteroides vulgatus* provirus prediction made by VIBRANT. The presence of viral hallmark protein, integrase and genome replication proteins strongly suggests this is an accurate prediction

In many environments, integrated proviruses can account for a substantial portion of the active viral community [75]. Despite this, few tools exist that are capable of identifying both lytic viruses from metagenomic scaffolds as well as proviruses that are integrated into host genomes. To account for this important group of viruses, VIBRANT identifies provirus regions within metagenomic scaffolds or whole genomes. VIBRANT is unique from most provirus prediction tools in that it does not rely on sequence motifs, such as integration sites, and therefore is especially useful for partial metagenomic scaffolds in which neither the provirus nor host region is complete. In addition, this functionality of VIBRANT provides the ability to trim non-viral (i.e., host genome) ends from viral scaffolds. This results in a more correct interpretation of genes that are encoded by the virus and not those that are misidentified as being within the viral genome region. Briefly, VIBRANT identifies proviruses by first identifying and isolating scaffolds and genomes at regions spanning several annotations with low v-scores. These regions were found to be almost exclusive to host genomes. After cutting the original sequence at these regions, a refinement step trims the putative provirus fragment to the first instance of a virus-like annotation to remove leftover host sequence (Fig. 4a). The final scaffold fragment is then analyzed by the neural network similar to non-excised scaffolds.

To assess VIBRANT’s ability to accurately extract provirus regions, we compared its performance to PHASTER and Prophage Hunter, two programs explicitly built for this task, as well as VirSorter. We compared the performance of these programs with VIBRANT on four complete bacterial genomes. VIBRANT and PHASTER predicted an equal number of proviruses, 17, while Prophage Hunter and VirSorter identified slightly less with 13 and 16 identifications, respectively (Fig. 4b). Only one putative provirus prediction (*Lactococcus lactis* putative provirus 6) was shared between all programs except VIBRANT. However, VIBRANT was able to identify two putative provirus regions (*Desulfovibrio vulgaris* putative provirus 7 and *Bacteroides vulgatus* putative provirus 1) that neither PHASTER nor Prophage Hunter identified, though VirSorter identified these likely due to the similar approach of extracting provirus regions. Manual inspection of the putative *Bacteroides vulgatus* provirus identified a number of virus hallmark and virus-like proteins suggesting that it is an accurate prediction (Fig. 4c). Our results suggest that VIBRANT has the ability to accurately identify proviruses and, in some cases, can outperform other tools in this task.

Both VIBRANT and VirSorter identify integrated proviruses from metagenomic assemblies by cutting host scaffolds at either end of a provirus region. By employing this method, these programs generate a more comprehensive understanding of a virome, but errors in identified cut sites may occur due to the diversity of genomic arrangements in both virus and host. This will result in fragmented viral genomes that should have remained intact. We assessed the error rate of VIBRANT and VirSorter (using virome decontamination mode) for cutting viral genomes. A total of 2466 *Caudovirales* complete genomes were acquired from the NCBI RefSeq database, including 74 megaphages with genomes greater than 200 kb. In total, VIBRANT fragmented 5 genomes whereas VirSorter fragmented 159 (categories 1 and 2) or 160 (categories 1, 2, and 3). Although relatively comparable, VirSorter incorrectly cut 6.2% more complete viral genomes compared to VIBRANT (6.4% versus 0.2%, respectively).

### Evaluating quality and completeness of predicted viral sequences

Determination of quality, in relation to completeness, of a predicted viral sequence has been notoriously difficult due to the absence of universally conserved viral genes. To date, the most reliable metric of completeness for metagenome- assembled viruses is to identify circular sequences (i.e., complete circular genomes). Therefore, the remaining alternatives rely on estimation based on encoded proteins that function in central viral processes: replication of genomes and assembly of new viral particles.

**Fig. 5:**
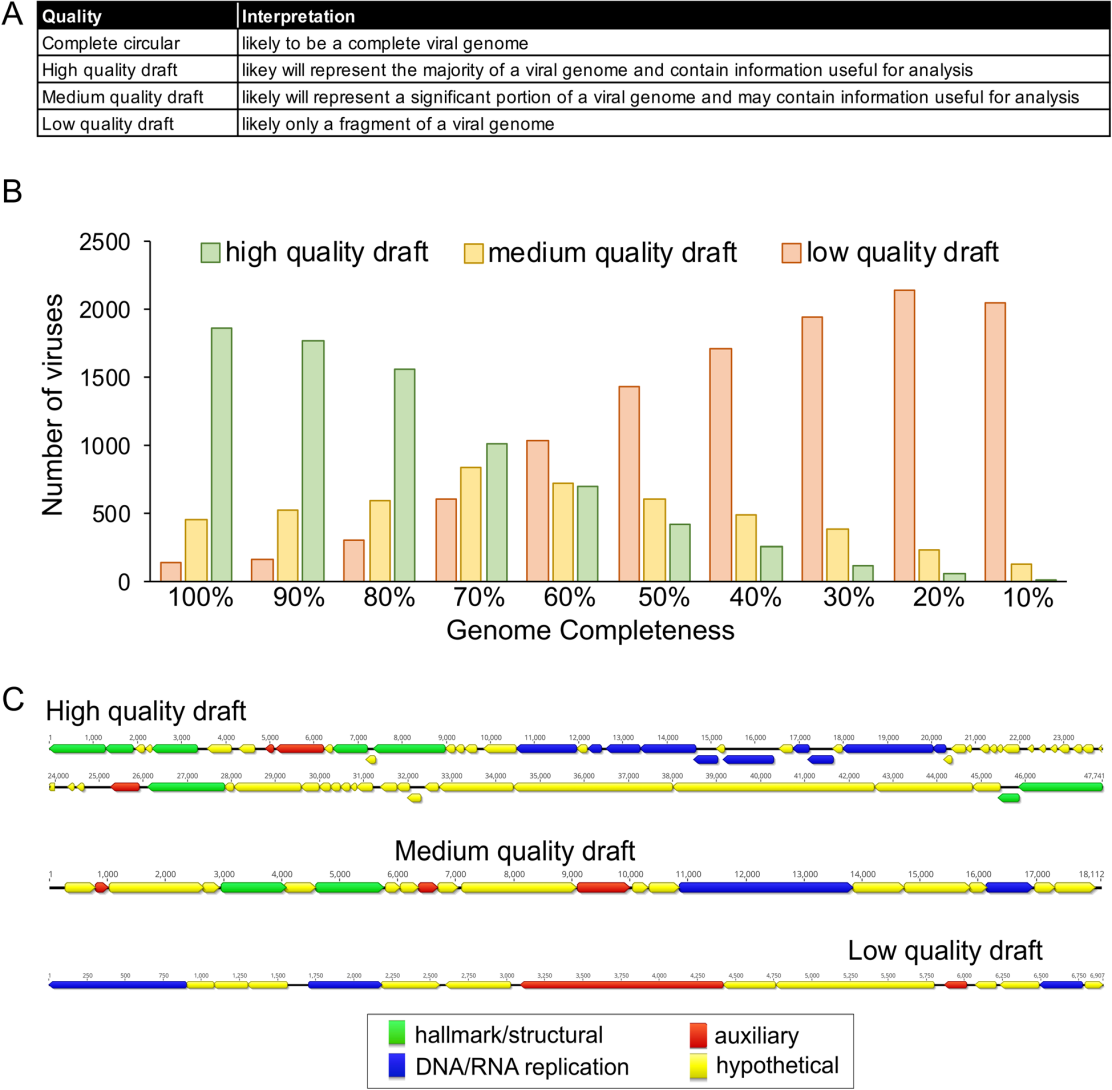
Estimation of genome quality of identified viral scaffolds. **a** Explanation of interpretation of quality categories: complete circular, high-quality draft, medium-quality draft, and low-quality draft. Quality generally represents total proteins, viral annotations, viral hallmark protein, and nucleotide replication proteins, which are common metrics used for manual verification of viral genomes. **b** Application of quality metrics to 2466 NCBI RefSeq *Caudovirales* viruses with decreasing genome completeness from 100 to 10% completeness, respective of total sequence length. All 2466 viruses are represented within each completeness group. **c** Examples of viral scaffolds representing low-, medium-, and high-quality draft categories

VIBRANT estimates the quality of predicted viral sequences, a relative proxy for completeness, and indicates sequences that are circular. To do this, VIBRANT uses annotation metrics of nucleotide replication and viral hallmark proteins. Hallmark proteins are those typically specific to viruses and those that are required for productive infection, such as structural (e.g., capsid, tail, baseplate), terminase, or viral holin/lysin proteins. Nucleotide replication proteins are a variety of proteins associated with either replication or metabolism, such as nucleases, polymerases, and DNA/RNA binding proteins. Viruses are categorized as low-, medium-, or high-quality draft as determined by VOG annotations (Fig. 5a, Additional File 17: Table S16). High-quality draft represents sequences that are likely to contain the majority of a virus’s complete genome and will contain annotations that are likely to aid in analysis of the virus, such as phylogenetic relationships and true positive verification. Medium draft quality represents the majority of a complete viral genome but is more likely to be a smaller portion in comparison to high quality. These sequences may contain annotations useful for analysis but are under less strict requirements compared to high quality. Finally, low draft quality constitutes sequences that were not found to be of high or medium quality. Many metagenomic scaffolds will likely be low-quality genome fragments, but this quality category may still contain the higher quality genomes of some highly divergent viruses.

We benchmarked VIBRANT’s viral genome quality estimation using a total of 2466 *Caudovirales* genomes from NCBI RefSeq database. Genomes were evaluated either as complete sequences or by removing 10% of the sequence at a time stepwise between 100 and 10% completeness (Fig. 5b). The results of VIBRANT’s quality analysis displayed a linear trend in indicating more complete genomes as high quality and less complete genomes as lower quality. The transition from categorizing genomes as high quality to medium quality ranged from 60 to 70% completeness. Although we acknowledge that VIBRANT’s metrics are not perfect, we demonstrate the first benchmarked approach to quantify and characterize genome quality associated with completeness of viral sequences. Manual inspection and visual verification of viral genomes that were characterized into each of these genome quality categories showed that quality estimations matched annotations (Fig. 5c).

### Identifying function in viral communities: metabolic analysis

Viruses are a dynamic and key facet in the metabolic networks of microbial communities and can reprogram the landscape of host and community metabolism during infection. This can often be achieved by modulating host metabolic networks through expression of AMGs encoded on viral genomes. Identifying these AMGs and their associated role in the function of communities is imperative for understanding complex microbiome dynamics, or in some cases can be used to predict virus-host relationships. VIBRANT is optimized for the evaluation of viral community function by identifying and classifying the metabolic capabilities encoded by a virome. To do this, VIBRANT identifies AMGs and assigns them into specific metabolic pathways and broader categories as designated by KEGG annotations.

To highlight the utility of this feature, we compared the metabolic function of IMG/VR viruses derived from several diverse environments: freshwater, marine, soil, human-associated, and city (Additional File 16: Figure S2). We found natural environments (freshwater, marine, and soil) to display a different pattern of metabolic capabilities compared to human environments (human-associated and city). Viruses originating from natural environments tend to largely encode AMGs for amino acid and cofactor/vitamin metabolism with a more secondary focus on carbohydrate and glycan metabolism. On the other hand, AMGs from city and human environments are dominated by amino acid metabolism, and to some extent cofactor/vitamin and sulfur relay metabolism. In addition to this broad distinction, all five environments appear slightly different from each other. Despite freshwater and marine environments appearing similar in the ratio of AMGs by metabolic category, the overlap in specific AMGs is less extensive. The dissimilarity between natural and human environments is likewise corroborated by the relatively low overlap in individual AMGs.

A useful observation provided by VIBRANT’s metabolic analysis is that there appears to be globally conserved AMGs (i.e., present within at least 10 of the 12 environments tested). These 14 genes—*dcm*, *cysH*, *folE*, *phnP*, *ubiG*, *ubiE*, *waaF*, *moeB*, *ahbD*, *cobS*, *mec*, *queE*, *queD*, *queC*—likely perform functions that are central to viral replication regardless of host or environment. Notably, *folE*, *queD*, *queE*, and *queC* constitute the entire 7-cyano-7-deazaguanine (preQ_0_) biosynthesis pathway, but the remainder of queuosine biosynthesis are entirely absent with the exception of *queF*. Certain AMGs are unique in that they are the only common representatives of a pathway among all AMGs identified, such as *phnP* for methylphosphonate degradation. These AMGs may indicate an evolutionary advantage for manipulating a specific step of a pathway, such as overcoming a reaction bottleneck, as opposed to modulating an entire pathway as seen with preQ_0_ biosynthesis. However, it should be noted that this list of 14 globally conserved AMGs may not be entirely inclusive of the core set of AMGs in a given environment.

**Fig. 6:**
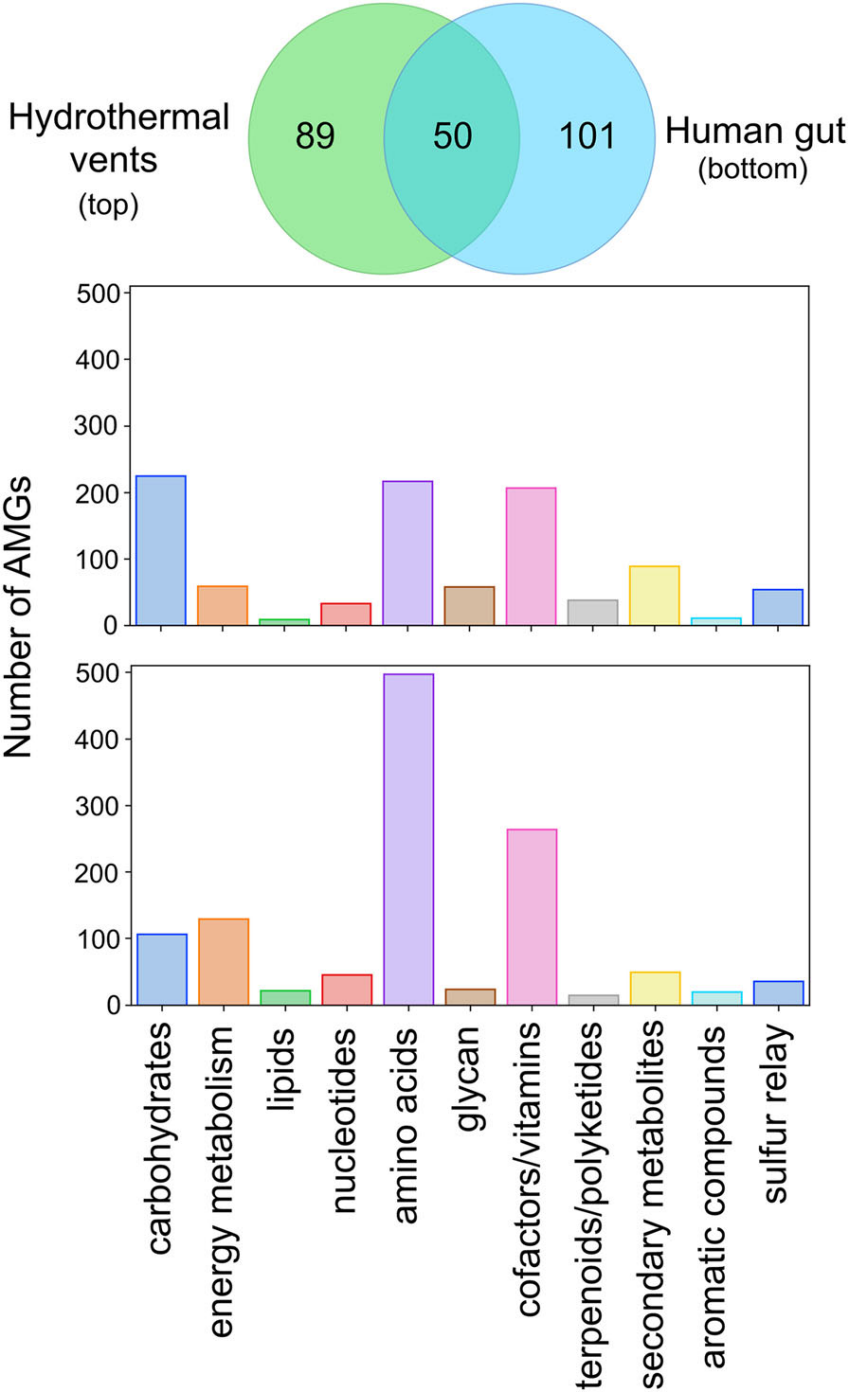
Comparison of AMG metabolic categories between hydrothermal vents and human gut. Venn diagram depicts the unique and shared non-redundant AMGs between 6 hydrothermal vent and 15 human gut metagenomes. The graphs depict the differential abundance of KEGG metabolic categories of respective AMGs for hydrothermal vents (top) and human gut (bottom)

VIBRANT was evaluated for its ability to provide new insights into viral community function by highlighting AMGs from mixed metagenomes. Using only data from VIBRANT’s direct outputs, we compared the viral metabolic profiles of 6 hydrothermal vent and 15 human gut metagenomes (Fig. 6). As anticipated, based on IMG/VR environment comparisons, the metabolic capabilities between the two environments were different even though the number of unique AMGs was relatively equal (138 for hydrothermal vents and 151 for human gut). The pattern displayed by metabolic categories for each metagenome was similar to that displayed by marine and human viromes. For hydrothermal vents, the dominant AMGs were part of carbohydrate, amino acid, and cofactor/vitamin metabolism, whereas human gut AMGs were mostly components of amino acid and, to some extent, cofactor/vitamin metabolism. Although the observed AMGs and metabolic pathways were overall different, about a third (50 total AMGs) of all AMGs from each environment was shared; between these metagenomes alone, all 14 globally conserved AMGs were present.

Observations of individual AMGs provided insights into how viruses interact within different environments. For example, tryptophan 7-halogenase (*prnA*) was identified in high abundance (45 total AMGs) within hydrothermal vent metagenomes but was absent from the human gut. Verification using GOV2 (Global Ocean Viromes 2.0) [76] and Human Gut Virome databases supported our finding that *prnA* appears to be constrained to aquatic environments, which is further supported by the gene’s presence on several marine cyanophages. PrnA catalyzes the initial reaction for the formation of pyrrolnitrin, a strong antifungal antibiotic. Identification of this AMG only within aquatic environments suggests a directed role in aquatic virus lifestyles. Similarly, cysteine desulfhydrase (*iscS*) was abundant (14 total AMGs) within the human gut metagenomes but not hydrothermal vents.

### Application of VIBRANT: identification of viruses from individuals with Crohn’s disease

We applied VIBRANT to identify viruses of at least 5 kb in length from 102 human gut metagenomes (discovery dataset): 49 from individuals with Crohn’s disease and 53 from healthy individuals [58, 59]. VIBRANT identified 14,121 viruses out of 511,977 total scaffolds. These viral sequences were dereplicated to 8822 non-redundant viral sequences using a cutoff of 95% nucleotide identity over at least 70% of the sequence. We next used read coverage of each virus sequence from all 102 metagenomes to calculate relative differential abundance across Crohn’s disease and healthy individuals. In total, we found 721 viral sequences to be more abundant in the gut microbiomes associated with Crohn’s disease (Crohn’s-associated) and 950 to be more abundant in healthy individuals (healthy-associated).

Using these viruses identified by VIBRANT, we sought to identify taxonomic or host-association relationships to differentiate the viral communities of individuals with Crohn’s disease. We used vConTACT2 to cluster the 721 Crohn’s- or 950 healthy-associated virus sequences with reference genomes using protein similarity. The majority of virus sequences (95.5%) were not clustered with any reference genome at approximately the genus level suggesting VIBRANT may have identified a large pool of novel or unique viral genomes. Although fewer total viruses were associated with Crohn’s disease, significantly more were clustered to at least one representative at the genus level (72 for Crohn’s and 4 for healthy). Interestingly, no differentially abundant viruses from healthy individuals clustered with Enterobacterales-infecting reference viruses (enteroviruses), yet the majority (60/76) of Crohn’s-associated viruses were clustered with known enteroviruses, such as Lambda- and Shigella-related viruses. The remaining 16 viruses mainly clustered with *Caudovirales* infecting *Lactococcus*, *Clostridium*, *Riemerella*, *Klebsiella*, and *Salmonella* species, though *Microviridae* and a likely complete crAssphage were also identified. A significant proportion of all Crohn’s-associated viruses (250/721) and the majority of genus-level clustered viruses (42/76) were found to be integrated sequences within a microbial genomic scaffold but were able to be identified due to VIBRANT’s ability to excise proviruses.

**Fig. 7:**
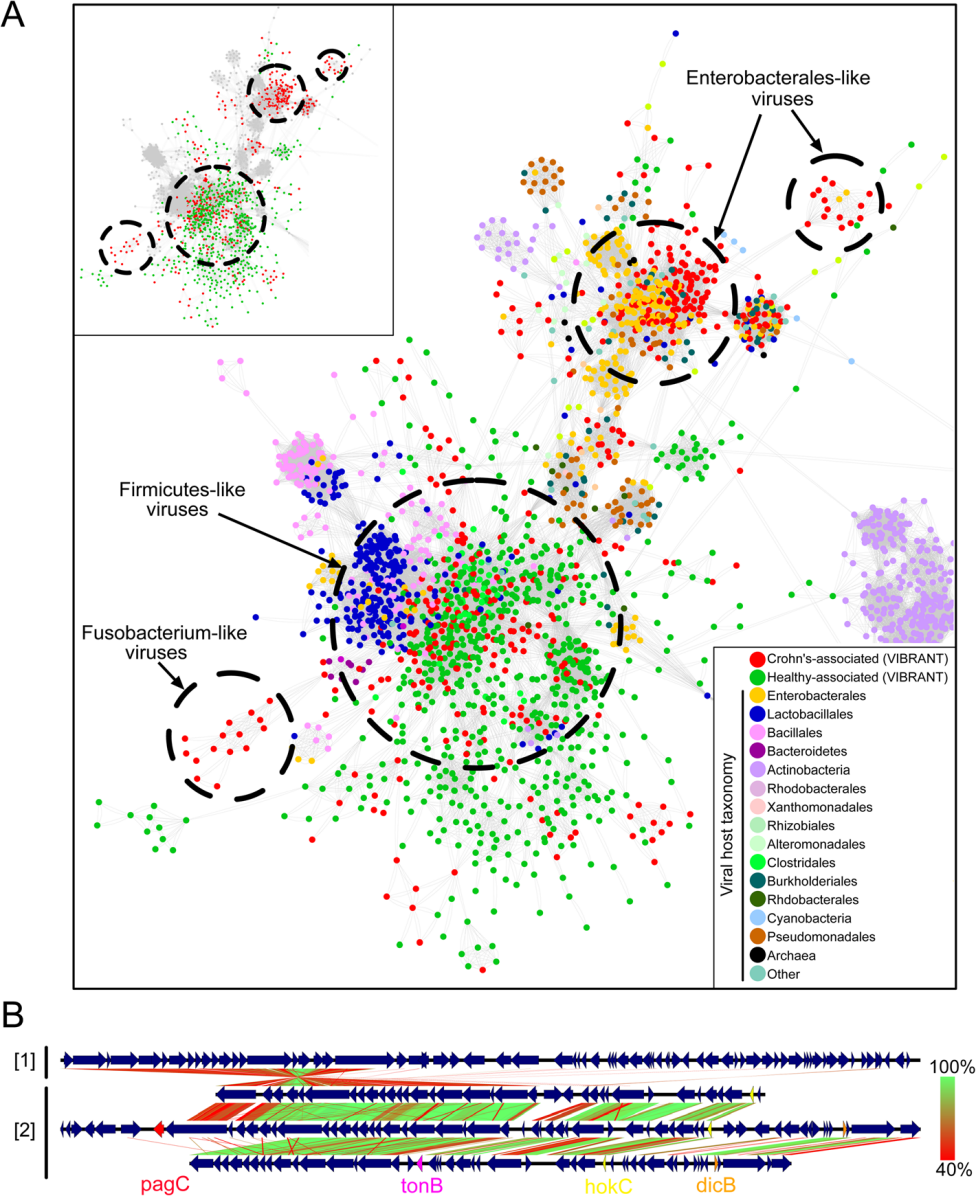
Viral metabolic comparison between Crohn’s disease and healthy individuals gut metagenomes. **a** Partial view of vConTACT2 protein network clustering of viruses identified by VIBRANT and reference viruses. Small clusters and clusters with no VIBRANT representatives are not shown. Each dot represents one genome and is colored according to host or dataset association. Relevant viral groups are indicated by dotted circles (circles enclose estimated boundaries). **b** tBLASTx similarity comparison between (1) Escherichia phage Lambda and (2) three Crohn’s-associated viruses identified by VIBRANT. Putative virulence genes are indicated as follows: *pagC*, *tonB*, *hokC*, and *dicB*

We also generated a protein sharing network containing all 721 Crohn’s and 950 healthy-associated virus sequences, which corresponded to taxonomic and host relatedness (Fig. 7a). This protein network identified two different clustering patterns: (1) overlapping Crohn’s and healthy-associated viral populations clustered with *Firmicutes*-like viruses which may be indicative of a stable gut viral community; (2) Crohn’s-associated viruses clustered with Enterobacterales-like and *Fusobacterium*-like viruses which may be indicative of a state of dysbiosis. The presence of a greater diversity and abundance of Enterobacterales and Fusobacteria has previously been linked to Crohn’s disease [77, 78], and therefore the presence of viruses infecting these bacteria may provide similar information.

VIBRANT provides annotation information for all of the identified viruses which can be used to infer functional characteristics in conjunction with host association. Comparison of Crohn’s-associated Lambda-like virus genomic content and arrangement suggested a possible role of virally encoded host-persistence and virulence genes that are absent in the healthy-associated virome (Fig. 7b). Among all Crohn’s-associated viruses, 17 total genes (*bor*, *dicB*, *dicC*, *hokC*, *kilR*, *pagC*, *ydaS*, *ydaT*, *yfdN*, *yfdP*, *yfdQ*, *yfdR*, *yfdS*, *yfdT*, *ymfL*, *ymfM*, and *tonB*) that have the potential to impact host survival or virulence were identified. Importantly, no healthy-associated viruses encoded such genes (Table 2). The presence of these putative dysbiosis-associated genes (DAGs) may contribute to the manifestation and/or persistence of disease, similar to what has been proposed for the bacterial microbiome [79–81]. For example, *pagC* encodes an outer membrane virulence factor associated with enhanced survival of the host bacterium within the gut [82]. The identification of *dicB* encoded on a putative *Escherichia* virus is unique in that it may represent a “cryptic” provirus that protects the host from lytic viral infection, thus likely to enhance the ability of the host to survive within the gut [83]. Finally, *hokC* may indicate mechanisms of virally encoded virulence [84].

**Table 2:** Identification of putative DAGs encoded by Crohn’s-associated viruses

ID	Gene	Name	Crohn’s disease	Healthy
PF06291.11	*bor*	Bor protein	8	0
K22304	*dicB*	Cell division inhibition protein	8	0
K22302	*dicC*	Transcriptional repressor of cell division inhibition gene dicB	18	0
K18919	*hokC*	Protein HokC/D	16	0
VOG11478	*kilR*	Killing protein	15	0
K07804	*pagC*	Putative virulence related protein	13	0
PF15943.5	*ydaS*	Putative antitoxin of bacterial toxin-antitoxin system	22	0
PF06254.11	*ydaT*	Putative bacterial toxin	18	0
VOG04806	*yfdN*	Uncharacterized protein	19	0
VOG01357	*yfdP*	Uncharacterized protein	11	0
VOG11472	*yfdQ*	Uncharacterized protein	11	0
VOG01639	*yfdR*	Uncharacterized protein	17	0
VOG01103	*yfdS*	Uncharacterized protein	18	0
VOG16442	*yfdT*	Uncharacterized protein	8	0
VOG00672	*ymfL*	Uncharacterized protein	25	0
VOG21507	*ymfM*	Uncharacterized protein	9	0
K03832	*tonB*	periplasmic protein	3	0

The differential abundance between Crohn’s disease and healthy metagenomes of 17 putative DAGs. Abundance of each gene represents non-redundant annotations or total gene copy number, from Crohn’s-associated and healthy-associated viruses

To characterize the distribution and association of DAGs with Crohn’s disease, we calculated differential abundance for two highly abundant DAG-encoding viruses across all metagenome samples. The first virus encoded *pagC* and *yfdN*, and the second encoded *dicB*, *dicC*, and *hokC*. Comparison of Crohn’s disease to healthy metagenomes indicates that these viruses are present within the gut metagenomes of multiple individuals but more abundant in association with Crohn’s disease (Additional File 16: Figure S3A). This suggests an association of disease with not only putative DAGs, but also specific and potentially persistent, viral groups that encode them. In order to correlate increased abundance with biological activity, we calculated the index of replication (iRep) for each of the two viruses [64]. Briefly, iRep is a function of differential read coverage which is able to provide an estimate of active genome replication. Seven metagenomes containing the greatest abundance for each virus were selected for iRep analysis and indicated that each virus was likely active at the time of collection (Additional File 16: Figure S3B).

To validate these aforementioned findings, we applied VIBRANT to two additional metagenomic datasets from cohorts of individuals with Crohn’s disease and healthy individuals (validation dataset): 43 from individuals with Crohn’s disease and 21 from healthy individuals [66, 67]. VIBRANT identified 3759 redundant viral genomes from Crohn’s-associated metagenomes and 1444 from healthy-associated metagenomes. Determination of protein networks and visualization similarly identified clustering of Crohn’s-associated viruses with reference enteroviruses (Additional File 16: Figure S4). Likewise, we were able to identify 15 out of the 17 putative DAGs to be present in higher abundance in the Crohn’s disease microbiome (Additional File 18: Table S17). This validates our findings of the presence of unique viruses and proteins associated with Crohn’s disease and suggests that Enterobacterales-like viruses and putative DAGs may act as markers of Crohn’s disease. Overall, our results suggest that VIBRANT provides a platform for characterizing these relationships.

## Discussion

Viruses that infect bacteria and archaea are key components in the structure, dynamics, and interactions of microbial communities [2, 6, 10, 76, 85]. Tools that are capable of efficient recovery of these viral genomes from mixed metagenomic samples are likely to be fundamental to the growing applications of metagenomic sequencing and analyses. Importantly, such tools would need to reduce bias associated with specific viral groups (e.g., *Caudovirales*) and highly represented environments (e.g., marine). Moreover, viruses that exist as integrated proviruses within host genomes should not be ignored as they can represent a substantial fraction of infections in certain conditions and also persistent infections within a community [75].

Here we have presented VIBRANT, a newly described method for the automated recovery of both free and integrated viral genomes from metagenomes that hybridizes neural network machine learning and protein signatures. VIBRANT utilizes metrics of non-reference-based protein similarity annotation from KEGG, Pfam, and VOG databases in conjunction with a unique “v-score” metric to recover viruses with little to no biases. VIBRANT was built with the consideration of the human guided intuition used to manually inspect metagenomic scaffolds for viral genomes and packages these ideas into an automated software. This platform originates from the notion that proteins generally considered as non-viral, such as ribosomal proteins [86], may be decidedly common among viruses and should be considered accordingly when viewing annotations. V-scores are meant to provide a quantitative metric for the level of virus-association for each annotation used by VIBRANT, especially for Pfam and KEGG HMMs. That is, v-scores provide a means for both highlighting common or hallmark viral proteins as well as differentiating viral from non-viral annotations. In addition, v-scores give a quantifiable value to viral hallmark genes instead of categorizing them in a binary fashion.

VIBRANT was not only built for the recovery of viral genomes, but also to act as a platform for investigating the function of a viral community. VIBRANT supports the analysis of viromes by assembling useful annotation data and categorizing the metabolic pathways of viral AMGs. Using annotation signatures, VIBRANT furthermore is capable of estimating genome quality and distinguishing between lytic and lysogenic viruses. To our knowledge, VIBRANT is the first software that integrates virus identification, annotation, and estimation of genome completeness into a stand-alone program.

Benchmarking and validation of VIBRANT indicated improved performance compared to VirSorter [37], VirFinder [41], and MARVEL [45], three commonly used programs for identifying viruses from metagenomes. This included a substantial increase in the relationship between true virus identifications (recall, true positive rate) and false non-virus identifications (specificity, false positive rate). That is, VIBRANT recovered more viruses with no discernable expense to false identifications. The result was that VIBRANT was able to recover an average of 2.3 and 1.7 more viral sequence from real metagenomes than VirFinder and VirSorter, respectively. When tested on metagenome-assembled viral genomes from IMG/VR [87] representing diverse environments, VIBRANT was found to have no perceivable environment bias towards identifying viruses. In comparison to provirus prediction tools, specifically PHASTER [47], Prophage Hunter [48], and VirSorter, VIBRANT was shown to be proficient in identifying viral regions within bacterial genomes. This included the identification of a putative *Bacteroides* provirus that PHASTER and Prophage Hunter were unable to identify. The importance of integrated provirus prediction was underscored in the analysis of Crohn’s disease metagenomes since it was found that a significant proportion of disease-related viruses were temperate viruses existing as host-integrated genomes.

VIBRANT’s method allows for the distinction between scaffold size and coding capacity in designating the minimum length of virus identifications. Traditionally, a cutoff of 5000 bp has been used to filter for scaffolds of a sufficient length for analysis. This is under the presumption that a longer sequence will be likely to encode more proteins. For example, this cutoff has been adopted by IMG/VR. However, we suggest a total protein cutoff of four open reading frames rather than sequence length cutoff to be more suitable for comprehensive characterization of the viral community. VIBRANT’s method works as a strict function of total encoded proteins and is completely agnostic to sequence length for analysis. Therefore, the boundary of minimum encoded proteins will support a more guided cutoff for quality control of virus identifications. For example, increasing the minimum sequence length to 5000 bp will have no effect on accuracy or ability to recall viruses since VIBRANT will only be considerate of the minimum total proteins, which is set to four. The result will be the loss of all 1000 to 4999 bp viruses that still encode at least four proteins. To visualize this distinction, we applied VIBRANT with various length cutoffs to the previously used estuary virome (see Table 1). Input sequences were stepwise limited from 1000 to 10,000 bp (1000 bp steps) or four open reading frames to 13 open reading frames (one open reading frame steps) in length. Limiting to open reading frames indicated a reduced drop-off in total virus identifications and total viral sequence compared to a minimum sequence length limit (Additional File 16: Figure S5).

The output data generated by VIBRANT—protein/gene annotation information, protein/gene sequences, HMM scores and e-values, viral sequences in FASTA and GenBank format, indication of AMGs, genome quality, etc.—provides a platform for easily replicated pipeline analyses. Application of VIBRANT to characterize the function of Crohn’s-associated viruses emphasizes this utility. VIBRANT was not only able to identify a substantial number of viral genomes, but also provided meaningful information regarding putative DAGs, viral sequences for differential abundance calculation and genome alignment, viral proteins for clustering, and AMGs for metabolic comparisons.

## Conclusions

Our construction of the VIBRANT platform expands the current potential for virus identification and characterization from metagenomic and genomic sequences. When compared to two widely used software programs, VirFinder and VirSorter, we show that VIBRANT improves total viral sequence and protein recovery from diverse human and natural environments. As sequencing technologies improve and metagenomic datasets contain longer sequences, VIBRANT will continue to outcompete programs built for short scaffolds (e.g., 500–3000 bp) by identifying more higher quality genomes. Our workflow, through the annotation of viral genomes, aids in the capacity to discover how viruses of bacteria and archaea may shape an environment, such as driving specific metabolism during infection or dysbiosis in the human gut. Furthermore, VIBRANT is the first virus identification software to incorporate annotation information into the curation of predictions, estimation of genome quality, and infection mechanism (i.e., lytic vs lysogenic). We anticipate that the incorporation of VIBRANT into microbiome analyses will provide easy interpretation of viral data, enabled by VIBRANT’s comprehensive functional analysis platform and visualization of information.

## Supplementary information

**Additional File 1: Table S1.** List of NCBI accession numbers for bacterial and archaeal genomes, plasmids, and viral genomes used in this study.

**Additional File 2: Table S2.** Number and sizes of sequence fragments used to train and test VIBRANT for viruses, plasmids, and bacteria and archaea.

**Additional File 3: Table S3.** List of all HMM names used by VIBRANT.

**Additional File 4: Table S4.** List of all KEGG, Pfam and VOG annotation names and associated v-scores (if greater than zero).

**Additional File 5: Table S5.** Unparsed HMM table output from KEGG annotations used to generate KEGG v-scores.

**Additional File 6: Table S6.** Unparsed HMM table output from Pfam annotations used to generate Pfam v-scores.

**Additional File 7: Table S7.** Unparsed HMM table output from VOG annotations used to generate VOG v-scores.

**Additional File 8: Table S8.** Normalized data used to train the neural network machine learning classifier.

**Additional File 9: Table S9.** Normalized data used to test the neural network machine learning classifier.

**Additional File 10: Table S10.** Equations used for benchmarking analyses.

**Additional File 11: Table S11.** Calculations and results of benchmarking analyses for VIBRANT, VirSorter, VirFinder and MARVEL.

**Additional File 12: Table S12.** List of all KEGG annotations determined as AMGs.

**Additional File 13: Table S13.** Lists of KEGG, Pfam and VOG annotations used to generate annotation metrics for neural network classification. Designated by asterisks are the lists of all VOG annotations determined as nucleotide replication-associated or viral hallmark-associated, which are used during prediction and quality estimation.

**Additional File 14: Table S14.** Results from DESeq2 analysis for 8,789 non-redundant viruses from the Crohn’s Disease discovery dataset.

**Additional File 15: Table S15.** Complete VIBRANT-derived annotations of validated viral scaffolds that encode putative DAGs, respective of Table 2.

**Additional File 16: **Supplemental Methods and Supplemental **Figures S1-S5.**

**Additional File 17: Table S16.** Number of *Caudovirales* genomes and genomic fragments identified per quality estimation category, exact rules used to estimate genome quality and the interpretation of quality estimations.

**Additional File 18: Table S17.** Validation of the greater abundance of viral DAGs in individuals with Crohn’s Disease compared to healthy individuals.

**Additional File 19: Table S18.** Description of set cutoffs implemented before neural network machine learning analysis for KEGG and Pfam annotations.

**Additional File 20: Table S19.** List of datasets used from He *et al.*, Ijaz *et al.* and Gevers *et al.*

**Additional File 21: Table S20.** VIBRANT runtimes and resource requirements for datasets of various sizes and compositions.

## Data Availability

VIBRANT is implemented in Python and all scripts and associated files are freely available at https://github.com/AnantharamanLab/VIBRANT/. The datasets supporting the conclusions of this article are included within the article and its additional files (Additional File 1: Table S1 and Additional File 20: Table S19). VIBRANT is also freely available for use as an application through the CyVerse Discovery Environment; to use the application visit https://de.cyverse.org/de/. Additional details of relevant data are available from the corresponding author on request.

## References

[1] Breitbart M, Rohwer F (2005). Here a virus, there a virus, everywhere the same virus?. Trends in Microbiology..

[2] Wommack KE, Colwell RR (2000). Virioplankton: viruses in aquatic ecosystems. Microbiol Mol Biol Rev..

[3] Danovaro R, Serresi M (2000). Viral density and virus-to-bacterium ratio in deep-sea sediments of the Eastern Mediterranean. Appl Environ Microbiol..

[4] Suttle CA (2007). Marine viruses — major players in the global ecosystem. Nature Reviews Microbiology..

[5] Heldal M, Bratbak G (1991). Production and decay of viruses in aquatic environments. Mar Ecol Prog Ser..

[6] Gobler CJ, Hutchins DA, Fisher NS, Cosper EM, Saňudo-Wilhelmy SA (1997). Release and bioavailability of C, N, P Se, and Fe following viral lysis of a marine chrysophyte. Limnology and Oceanography..

[7] Jiao N, Herndl GJ, Hansell DA, Benner R, Kattner G, Wilhelm SW (2010). Microbial production of recalcitrant dissolved organic matter: long-term carbon storage in the global ocean. Nature Reviews Microbiology..

[8] Brussaard CPD, Wilhelm SW, Thingstad F, Weinbauer MG, Bratbak G, Heldal M (2008). Global-scale processes with a nanoscale drive: the role of marine viruses. The ISME Journal..

[9] Fuhrman JA (1999). Marine viruses and their biogeochemical and ecological effects. Nature..

[10] Wilhelm SW, Suttle CA (1999). Viruses and nutrient cycles in the sea. BioScience..

[11] Norman JM, Handley SA, Baldridge MT, Droit L, Liu CY, Keller BC (2015). Disease-specific alterations in the enteric virome in inflammatory bowel disease. Cell..

[12] Barr JJ (2019). Missing a phage: unraveling tripartite symbioses within the human gut. mSystems.

[13] Barr JJ, Auro R, Furlan M, Whiteson KL, Erb ML, Pogliano J (2013). Bacteriophage adhering to mucus provide a non-host-derived immunity. Proceedings of the National Academy of Sciences..

[14] Rohwer F (2003). Global phage diversity. Cell..

[15] Jiang SC, Paul JH (1998). Gene transfer by transduction in the marine environment. APPL ENVIRON MICROBIOL..

[16] Gregory AC, Solonenko SA, Ignacio-Espinoza JC, LaButti K, Copeland A, Sudek S (2016). Genomic differentiation among wild cyanophages despite widespread horizontal gene transfer. BMC Genomics..

[17] Sanjuán R, Nebot MR, Chirico N, Mansky LM, Belshaw R (2010). Viral mutation rates. J Virol..

[18] Kim B, Kim ES, Yoo Y-J, Bae H-W, Chung I-Y, Cho Y-H. Phage-derived antibacterials: harnessing the simplicity, plasticity, and diversity of phages. Viruses [Internet]. 2019 [cited 2019 Oct 24];11. Available from: https://www.ncbi.nlm.nih.gov/pmc/articles/PMC6466130/. 10.3390/v11030268 PMC6466130 30889807

[19] Peng S-Y, You R-I, Lai M-J, Lin N-T, Chen L-K, Chang K-C (2017). Highly potent antimicrobial modified peptides derived from the Acinetobacter baumannii phage endolysin LysAB2. Sci Rep..

[20] Holt A, Cahill J, Ramsey J, O’Leary C, Moreland R, Martin C, et al. Phage-encoded cationic antimicrobial peptide used for outer membrane disruption in lysis. bioRxiv. 2019;515445.

[21] Harada LK, Silva EC, Campos WF, Del Fiol FS, Vila M, Dąbrowska K (2018). Biotechnological applications of bacteriophages: state of the art. Microbiological Research..

[22] Sharma RS, Karmakar S, Kumar P, Mishra V (2019). Application of filamentous phages in environment: a tectonic shift in the science and practice of ecorestoration. Ecology and Evolution..

[23] Bragg JG, Chisholm SW (2008). Modeling the fitness consequences of a cyanophage-encoded photosynthesis gene. PLOS ONE..

[24] Mann NH, Cook A, Millard A, Bailey S, Clokie M (2003). Bacterial photosynthesis genes in a virus. Nature..

[25] Anantharaman K, Duhaime MB, Breier JA, Wendt KA, Toner BM, Dick GJ (2014). Sulfur oxidation genes in diverse deep-sea viruses. Science..

[26] Emerson JB, Roux S, Brum JR, Bolduc B, Woodcroft BJ, Jang HB (2018). Host-linked soil viral ecology along a permafrost thaw gradient. Nature Microbiology..

[27] Trubl G, Jang HB, Roux S, Emerson JB, Solonenko N, Vik DR (2018). Soil viruses are underexplored players in ecosystem carbon processing. mSystems.

[28] Waldbauer JR, Coleman ML, Rizzo AI, Campbell KL, Lotus J, Zhang L (2019). Nitrogen sourcing during viral infection of marine cyanobacteria. PNAS..

[29] Stent GS, Maaløe O (1953). Radioactive phosphorus tracer studies on the reproduction of T4 bacteriophage: II. Kinetics of phosphorus assimilation. Biochimica et Biophysica Acta..

[30] Kozloff LM, Knowlton K, Putnam FW, Evans EA (1951). Biochemical studies of virus reproduction V. the origin of bacteriophage nitrogen. J Biol Chem..

[31] Thompson LR, Zeng Q, Kelly L, Huang KH, Singer AU, Stubbe J (2011). Phage auxiliary metabolic genes and the redirection of cyanobacterial host carbon metabolism. PNAS..

[32] Breitbart M, Thompson L, Suttle C, Sullivan M (2007). Exploring the vast diversity of marine viruses. Oceanography..

[33] Hurwitz BL, Hallam SJ, Sullivan MB (2013). Metabolic reprogramming by viruses in the sunlit and dark ocean. Genome Biology..

[34] Roux S, Hawley AK, Beltran MT, Scofield M, Schwientek P, Stepanauskas R (2014). Ecology and evolution of viruses infecting uncultivated SUP05 bacteria as revealed by single-cell- and meta-genomics. eLife Sciences..

[35] Labonté JM, Swan BK, Poulos B, Luo H, Koren S, Hallam SJ (2015). Single-cell genomics-based analysis of virus–host interactions in marine surface bacterioplankton. ISME J..

[36] Trubl G, Solonenko N, Chittick L, Solonenko SA, Rich VI, Sullivan MB (2016). Optimization of viral resuspension methods for carbon-rich soils along a permafrost thaw gradient. PeerJ..

[37] Roux S, Enault F, Hurwitz BL, Sullivan MB (2015). VirSorter: mining viral signal from microbial genomic data. PeerJ..

[38] Wommack KE, Bhavsar J, Polson SW, Chen J, Dumas M, Srinivasiah S (2012). VIROME: a standard operating procedure for analysis of viral metagenome sequences. Standards in Genomic Sciences..

[39] Roux S, Faubladier M, Mahul A, Paulhe N, Bernard A, Debroas D (2011). Metavir: a web server dedicated to virome analysis. Bioinformatics..

[40] El-Gebali S, Mistry J, Bateman A, Eddy SR, Luciani A, Potter SC (2019). The Pfam protein families database in 2019. Nucleic Acids Res..

[41] Ren J, Ahlgren NA, Lu YY, Fuhrman JA, Sun F (2017). VirFinder: a novel k-mer based tool for identifying viral sequences from assembled metagenomic data. Microbiome..

[42] Fang Z, Tan J, Wu S, Li M, Xu C, Xie Z, et al. PPR-Meta: a tool for identifying phages and plasmids from metagenomic fragments using deep learning. Gigascience [Internet]. 2019 [cited 2019 Aug 5];8. Available from: https://academic.oup.com/gigascience/article/8/6/giz066/5521157. 10.1093/gigascience/giz066 PMC6586199 31220250

[43] Ahlgren NA, Ren J, Lu YY, Fuhrman JA, Sun F (2017). Alignment-free $d_2^*$ oligonucleotide frequency dissimilarity measure improves prediction of hosts from metagenomically-derived viral sequences. Nucleic Acids Res..

[44] Ponsero AJ, Hurwitz BL. The promises and pitfalls of machine learning for detecting viruses in aquatic metagenomes. Front Microbiol [Internet]. 2019 [cited 2019 Oct 24];10. Available from: https://www.frontiersin.org/articles/10.3389/fmicb.2019.00806/full. 10.3389/fmicb.2019.00806 PMC6477088 31057513

[45] Amgarten D, Braga LPP, da Silva AM, Setubal JC. MARVEL, a tool for prediction of bacteriophage sequences in metagenomic bins. Front Genet [Internet]. 2018 [cited 2019 Aug 5];9. Available from: https://www.frontiersin.org/articles/10.3389/fgene.2018.00304/full. 10.3389/fgene.2018.00304 PMC6090037 30131825

[46] Zheng T, Li J, Ni Y, Kang K, Misiakou M-A, Imamovic L (2019). Mining, analyzing, and integrating viral signals from metagenomic data. Microbiome..

[47] Arndt D, Grant JR, Marcu A, Sajed T, Pon A, Liang Y (2016). PHASTER: a better, faster version of the PHAST phage search tool. Nucleic Acids Res..

[48] Song W, Sun H-X, Zhang C, Cheng L, Peng Y, Deng Z (2019). Prophage Hunter: an integrative hunting tool for active prophages. Nucleic Acids Res..

[49] Merchant N, Lyons E, Goff S, Vaughn M, Ware D, Micklos D (2016). The iPlant collaborative: cyberinfrastructure for enabling data to discovery for the life sciences. PLOS Biology..

[50] Hyatt D, Chen G-L, LoCascio PF, Land ML, Larimer FW, Hauser LJ (2010). Prodigal: prokaryotic gene recognition and translation initiation site identification. BMC Bioinformatics..

[51] Krishnamurthy SR, Janowski AB, Zhao G, Barouch D, Wang D (2016). Hyperexpansion of RNA bacteriophage diversity. PLOS Biology..

[52] Altschul SF, Gish W, Miller W, Myers EW, Lipman DJ (1990). Basic local alignment search tool. J Mol Biol..

[53] Eddy SR (1998). Profile hidden Markov models. Bioinformatics..

[54] Kanehisa M, Goto S (2000). KEGG: Kyoto Encyclopedia of Genes and Genomes. Nucleic Acids Res..

[55] Aramaki T, Blanc-Mathieu R, Endo H, Ohkubo K, Kanehisa M, Goto S, et al. KofamKOALA: KEGG ortholog assignment based on profile HMM and adaptive score threshold. bioRxiv. 2019:602110. 10.1093/bioinformatics/btz859 PMC7141845 31742321

[56] Fu L, Niu B, Zhu Z, Wu S, Li W (2012). CD-HIT: accelerated for clustering the next-generation sequencing data. Bioinformatics..

[57] Pedregosa F, Varoquaux G, Gramfort A, Michel V, Thirion B, Grisel O (2011). Scikit-learn: machine learning in python. Journal of Machine Learning Research..

[58] He Q, Gao Y, Jie Z, Yu X, Laursen JM, Xiao L (2017). Two distinct metacommunities characterize the gut microbiota in Crohn’s disease patients. Gigascience..

[59] Pasolli E, Asnicar F, Manara S, Zolfo M, Karcher N, Armanini F (2019). Extensive unexplored human microbiome diversity revealed by over 150,000 genomes from metagenomes spanning age, geography, and lifestyle. Cell.

[60] Ondov BD, Treangen TJ, Melsted P, Mallonee AB, Bergman NH, Koren S (2016). Mash: fast genome and metagenome distance estimation using MinHash. Genome Biology..

[61] Delcher AL (2002). Fast algorithms for large-scale genome alignment and comparison. Nucleic Acids Research..

[62] Langmead B, Salzberg SL (2012). Fast gapped-read alignment with Bowtie 2. Nat Methods..

[63] Love MI, Huber W, Anders S (2014). Moderated estimation of fold change and dispersion for RNA-seq data with DESeq2. Genome Biology..

[64] Brown CT, Olm MR, Thomas BC, Banfield JF (2016). Measurement of bacterial replication rates in microbial communities. Nature Biotechnology..

[65] Sullivan MJ, Petty NK, Beatson SA (2011). Easyfig: a genome comparison visualizer. Bioinformatics..

[66] Gevers D, Kugathasan S, Denson LA, Vázquez-Baeza Y, Van Treuren W, Ren B (2014). The treatment-naive microbiome in new-onset Crohn’s disease. Cell Host Microbe..

[67] Ijaz UZ, Quince C, Hanske L, Loman N, Calus ST, Bertz M (2017). The distinct features of microbial “dysbiosis” of Crohn’s disease do not occur to the same extent in their unaffected, genetically-linked kindred. PLoS ONE..

[68] Shannon P (2003). Cytoscape: a software environment for integrated models of biomolecular interaction networks. Genome Research..

[69] Kristensen DM, Waller AS, Yamada T, Bork P, Mushegian AR, Koonin EV (2013). Orthologous gene clusters and taxon signature genes for viruses of prokaryotes. Journal of Bacteriology..

[70] Grazziotin AL, Koonin EV, Kristensen DM (2017). Prokaryotic Virus Orthologous Groups (pVOGs): a resource for comparative genomics and protein family annotation. Nucleic Acids Res..

[71] Hendricks SP, Mathews CK (1997). Regulation of T4 phage aerobic ribonucleotide reductase: simultaneous assay of the four activities. J Biol Chem..

[72] Roux S, Adriaenssens EM, Dutilh BE, Koonin EV, Kropinski AM, Krupovic M (2019). Minimum information about an uncultivated virus genome (MIUViG). Nature Biotechnology..

[73] Bowers RM, Kyrpides NC, Stepanauskas R, Harmon-Smith M, Doud D, Reddy TBK (2017). Minimum information about a single amplified genome (MISAG) and a metagenome-assembled genome (MIMAG) of bacteria and archaea. Nature Biotechnology..

[74] Tucker KP, Parsons R, Symonds EM, Breitbart M (2011). Diversity and distribution of single-stranded DNA phages in the North Atlantic Ocean. ISME J..

[75] Payet JP, Suttle CA (2013). To kill or not to kill: the balance between lytic and lysogenic viral infection is driven by trophic status. Limnology and Oceanography..

[76] Gregory AC, Zayed AA, Conceição-Neto N, Temperton B, Bolduc B, Alberti A, et al. Marine DNA viral macro- and microdiversity from pole to pole. Cell [Internet]. 2019 [cited 2019 Apr 30]; Available from: http://www.sciencedirect.com/science/article/pii/S0092867419303411. 10.1016/j.cell.2019.03.040 PMC6525058 31031001

[77] Morgan XC, Tickle TL, Sokol H, Gevers D, Devaney KL, Ward DV (2012). Dysfunction of the intestinal microbiome in inflammatory bowel disease and treatment. Genome Biology..

[78] Strauss J, Kaplan GG, Beck PL, Rioux K, Panaccione R, Devinney R (2011). Invasive potential of gut mucosa-derived Fusobacterium nucleatum positively correlates with IBD status of the host. Inflamm Bowel Dis..

[79] Shreiner AB, Kao JY, Young VB (2015). The gut microbiome in health and in disease. Curr Opin Gastroenterol..

[80] Clemente JC, Ursell LK, Parfrey LW, Knight R (2012). The impact of the gut microbiota on human health: an integrative view. Cell..

[81] Minot SS, Willis AD (2019). Clustering co-abundant genes identifies components of the gut microbiome that are reproducibly associated with colorectal cancer and inflammatory bowel disease. Microbiome..

[82] Nishio M, Okada N, Miki T, Haneda T, Danbara H (2005). Identification of the outer-membrane protein PagC required for the serum resistance phenotype in Salmonella enterica serovar Choleraesuis. Microbiology (Reading, Engl).

[83] Ragunathan PT, Vanderpool CK. Cryptic-prophage-encoded small protein DicB protects Escherichia coli from phage infection by inhibiting inner membrane receptor proteins. Journal of Bacteriology [Internet]. 2019 [cited 2019 Nov 11];201. Available from: https://jb.asm.org/content/201/23/e00475-19. 10.1128/JB.00475-19 PMC6832061 31527115

[84] Rasko DA, Rosovitz MJ, Myers GSA, Mongodin EF, Fricke WF, Gajer P (2008). The pangenome structure of Escherichia coli: comparative genomic analysis of E. coli commensal and pathogenic isolates. Journal of Bacteriology..

[85] Jover LF, Effler TC, Buchan A, Wilhelm SW, Weitz JS (2014). The elemental composition of virus particles: implications for marine biogeochemical cycles. Nature Reviews Microbiology..

[86] Mizuno CM, Guyomar C, Roux S, Lavigne R, Rodriguez-Valera F, Sullivan MB (2019). Numerous cultivated and uncultivated viruses encode ribosomal proteins. Nature Communications..

[87] Paez-Espino D, Roux S, Chen I-MA, Palaniappan K, Ratner A, Chu K (2019). IMG/VR v.2.0: an integrated data management and analysis system for cultivated and environmental viral genomes. Nucleic Acids Res..

