# Supplementary material for: VIBRANT: automated recovery, annotation and curation of microbial viruses, and evaluation of viral community function from genomic sequences
Source: Microbiome. 2020 Jun 10;8:90. doi: 10.1186/s40168-020-00867-0 (PMC7288430; doi:10.1186/s40168-020-00867-0)
Supplement: Supplementary file 17 — Additional File 16: Supplemental Methods and Supplemental Figures S1-S5. [file 40168_2020_867_MOESM16_ESM.pdf]

Supplemental Material for

VIBRANT: Automated recovery, annotation and curation of microbial viruses, and evaluation of viral community function from genomic sequences

Kristopher Kieft, Zhichao Zhou, and Karthik Anantharaman

Supplemental Methods

Non-neural network steps and assembly of annotation metrics

VIBRANT utilizes several manually curated cutoffs in order to remove the bulk of non-virus input scaffolds before the neural network classifier is implemented. These steps result in the generation of 27 annotation-derived metrics that are used by the neural network classifier for virus identification, which is followed by additional manually set cutoffs to curate the results.

First, open reading frames are predicted by Prodigal (-p meta) or a user may input predicted proteins. These proteins are then annotated with the 10,033 KEGG-derived HMMs. Putative integrated provirus regions are extracted at this step by using sliding windows of either four or nine proteins at a time (step size = 1 protein). Within these windows, scaffolds are fragmented according to v-scores and total KEGG annotations. Within the 4-protein window, scaffolds can be cut if (1) there are 0-1 unannotated proteins, 3-4 proteins with a v-score of 0-0.02 and a combined v-score of less than 0.06, or (2) three consecutive proteins with a v-score of 0 (considered as a 3-protein sub-window). Scaffolds will also be cut using a 9-protein window if nine consecutive proteins are annotated. Finally, if the final two proteins on a scaffold each have a v-score of 0, the scaffold will be cut. Only scaffold fragments that contain at least 8 proteins are retained. Following provirus excision, several manually set cutoffs are used to remove obvious non-viral scaffolds. Briefly, this is done by removing scaffolds with a high density of KEGG annotations (e.g., over 70% if less than 15 proteins or over 50% if greater than 15 proteins) or a high number of annotations with a v-score of 0 (e.g., over 15 total). V-scores are also used such that a scaffold that may be removed for having a high density of KEGG annotations will be retained if the v-score meets a specific threshold (e.g., average of 0.2).

Scaffolds that are retained are subsequently annotated by the 17,929 Pfam HMMs. In a similar manner to KEGG, scaffolds meeting set cutoffs for density and v-scores of Pfam HMMs are either retained or removed. For example, scaffolds with less than 15 total or density under 60% Pfam annotations are retained; a scaffold will be retained if it has greater than 60% Pfam annotations as well as an average v-score of at least 0.15. For both KEGG and Pfam cutoffs, full details of every cutoff can be found in Additional File 19: Table S18.

Following the aforementioned cutoff steps approximately 75-85% of non-viral scaffolds are removed. At this point scaffolds are annotated by the 19,182 VOG HMMs. Using VOG annotations and v-scores, as well as v-scores from KEGG and Pfam, putative proviruses are trimmed to remove ends that may still contain host proteins. To do this, any scaffold previously cut is trimmed, at both ends, to either the first instance of a VOG annotation or the first v-score of at least 0.1 from KEGG or Pfam annotations.

Annotations from all three databases are used to assemble 27 metrics for the neural network classifier. Briefly the metrics for each scaffold individually are as follows: (1) total encoded proteins, (2) total KEGG annotations, (3) sum of KEGG v-scores, (4) total Pfam annotations, (5)

sum of Pfam v-scores, (6) total VOG annotations, (7) sum of VOG v-scores, (8) total KEGG integration related annotations (e.g., integrase), (9) total KEGG annotations with a v-score of zero, (10) total Pfam integration related annotations (e.g., integrase), (11) total Pfam annotations with a v-score of zero, (12) total VOG redoxin (e.g., glutaredoxin) related annotations, (13) total VOG non-integrase integration related annotations, (14) total VOG integrase annotations, (15) total VOG ribonucleotide reductase related annotations, (16) total VOG nucleotide replication (e.g., DNA polymerase) related annotations, (17) total KEGG nuclease (e.g., restriction endonuclease) related annotations, (18) total KEGG toxin/anti-toxin related annotations, (19) total VOG hallmark protein (e.g., capsid) annotations, (20) total proteins annotated by KEGG, Pfam and VOG, (21) total proteins annotated by Pfam and VOG only, (22) total proteins annotated by Pfam and KEGG only, (23) total proteins annotated by KEGG and VOG only, (24) total proteins annotated by KEGG only, (25) total proteins annotated by Pfam only, (26) total proteins annotated by VOG only, and (27) total unannotated proteins. A complete list of all annotations used to generate these metrics can be found in Additional File 13: Table S13. Non-annotation features such as gene density, average gene length and strand switching were not used because they were found to decrease performance of the neural network classifier despite being differentiating features between bacteria/archaea and viruses; viruses tend to have shorter genes, less intergenic space and strand switch less frequently. This decreased performance is likely due to several reasons, such as errors associated with protein prediction (e.g., missed open reading frame leading to a large "intergenic" gap) or that scaffolds, due to being fragmented genomes in most cases, behave differently than the genome as a whole. For example, genomic regions encoding for large structural proteins will have a higher average gene size, or a small window of virus proteins may have a greater average strand switching level compared to the whole genome.

Additional viral datasets and metagenomes

The Integrated Microbial Genomes and Viruses (IMG/VR) v2.0 database (accessed July 2019) [1,2] was downloaded and scaffolds originating from animal-associated, aquatic sediment, city, marine A (coastal, gulf, inlet, intertidal, neritic, oceanic, pelagic and strait), marine B (hydrothermal vent, volcanic and oil), deep subsurface, freshwater, human-associated, plant-associated, soil, wastewater and wetland environments were selected for analysis. Venn diagram visualization of virus predictions with this dataset was made using Matplotlib (v3.0.0) [3].

For evaluation of 1kb and 3kb fragments new subset datasets were generated. For viruses, all circular viruses (i.e., assumed to be complete) from IMG/VR freshwater and soil environments as well as the Human Gut Virome database [4] were split into 3kb and redundant 1kb fragments. Recall metrics for viruses were reported as the average from the three datasets (i.e., IMG/VR freshwater, IMG/VR soil and Human Gut Virome database). For bacterial/archaeal genomic and plasmid fragments, 13kb and 15kb fragments from the comprehensive test dataset were split into 3kb and redundant 1kb fragments.

For eukaryotic contamination, three likely contaminant genomic sequences were acquired from NCBI: Candida albicans SC5314 chromosome 1 (NC_032089.1), Naegleria gruberi strain NEG-M (ACER01000000.1), and Ostreococcus sp. SAG9 (VIBA01000000.1). These sequences were split into fragments ranging from 1kb to 15kb.

Several published, assembled metagenomes from IMG/VR representing diverse environments were selected for comparing VIBRANT, Virsorter and VirFinder (IMG taxon IDs: 3300005281, 3300017813 and 3300000439). Fifteen publicly available datasets from the human gut were assembled for assessing VIBRANT and comparing the three programs [5]. Reads can be

found under NCBI BioProject PRJEB7774 (ERR688591, ERR688590, ERR688509, ERR608507, ERR608506, ERR688584, ERR688587, ERR688519, ERR688512, ERR688508, ERR688634, ERR688618, ERR688515, ERR688513, ERR688505). Reads were trimmed using Sickle (v1.33) [6] and assembled using metaSPAdes (v3.12.0 65) [7] (--meta -k 21,33,55,77,99). For hydrothermal vents, six publicly available hydrothermal plume samples were derived from Guaymas Basin (one sample) and Eastern Lau Spreading Center (five samples). Reads can be found under NCBI BioProject PRJNA314399 (SRR3577362) and PRJNA234377 (SRR1217367, SRR1217459, SRR1217564, SRR1217566, SRR1217452, SRR1217567, SRR1217465, SRR1217462, SRR1217460, SRR1217463, SRR1217565). Reads were trimmed using Sickle and assembled using metaSPAdes (--meta -k 21,33,55,77,99). Details of assembly and processing are outlined in Zhou *et al*. [8]. For analysis of Crohn's Disease metagenomes by VIBRANT, publicly available metagenomes were used; the metagenomes were sequenced by He *et al*. [9], Ijaz *et al*. [10] and Gevers *et al*. [11], and assembled by Pasolli *et al*. [12] (Additional File 20: Table S19).

The computational resource requirements and associated runtimes for VIBRANT were assessed using datasets of various sizes and composition (Additional File 21: Table S20). VIBRANT was able to evaluate large datasets quickly since it was built for efficient parallelization across CPUs.

References

1. Paez-Espino D, Eloe-Fadrosh EA, Pavlopoulos GA, Thomas AD, Huntemann M, Mikhailova N, et al. Uncovering Earth's virome. Nature. 2016;536:425–30.

2. Paez-Espino D, Roux S, Chen I-MA, Palaniappan K, Ratner A, Chu K, et al. IMG/VR v.2.0: an integrated data management and analysis system for cultivated and environmental viral genomes. Nucleic Acids Res. 2019;47:D678–86.

3. Hunter JD. Matplotlib: A 2D graphics environment. Computing In Science & Engineering. 2007;9:90–5.

4. Gregory AC, Zablocki O, Howell A, Bolduc B, Sullivan MB. The human gut virome database. bioRxiv. 2019;655910.

5. Feng Q, Liang S, Jia H, Stadlmayr A, Tang L, Lan Z, et al. Gut microbiome development along the colorectal adenoma–carcinoma sequence. Nature Communications. 2015;6:1–13.

6. Joshi N, Fass J. Sickle: A sliding-window, adaptive, quality-based trimming tool for FastQ files [Internet]. 2011. Available from: https://github.com/najoshi/sickle

7. Nurk S, Meleshko D, Korobeynikov A, Pevzner PA. metaSPAdes: a new versatile metagenomic assembler. Genome Res. 2017;27:824–34.

8. Zhou Z, Tran PQ, Kieft K, Anantharaman K. Genome diversification in globally distributed novel marine Proteobacteria is linked to environmental adaptation. bioRxiv. 2019;814418.

9. He Q, Gao Y, Jie Z, Yu X, Laursen JM, Xiao L, et al. Two distinct metacommunities characterize the gut microbiota in Crohn's disease patients. Gigascience. 2017;6:1–11.

10. Ijaz UZ, Quince C, Hanske L, Loman N, Calus ST, Bertz M, et al. The distinct features of microbial "dysbiosis" of Crohn's disease do not occur to the same extent in their unaffected, genetically-linked kindred. PLoS ONE. 2017;12:e0172605.

11. Gevers D, Kugathasan S, Denson LA, Vázquez-Baeza Y, Van Treuren W, Ren B, et al. The treatment-naive microbiome in new-onset Crohn's disease. Cell Host Microbe. 2014;15:382–92.

12. Pasolli E, Asnicar F, Manara S, Zolfo M, Karcher N, Armanini F, et al. Extensive Unexplored Human Microbiome Diversity Revealed by Over 150,000 Genomes from Metagenomes Spanning Age, Geography, and Lifestyle. Cell. 2019;176:649-662.e20.

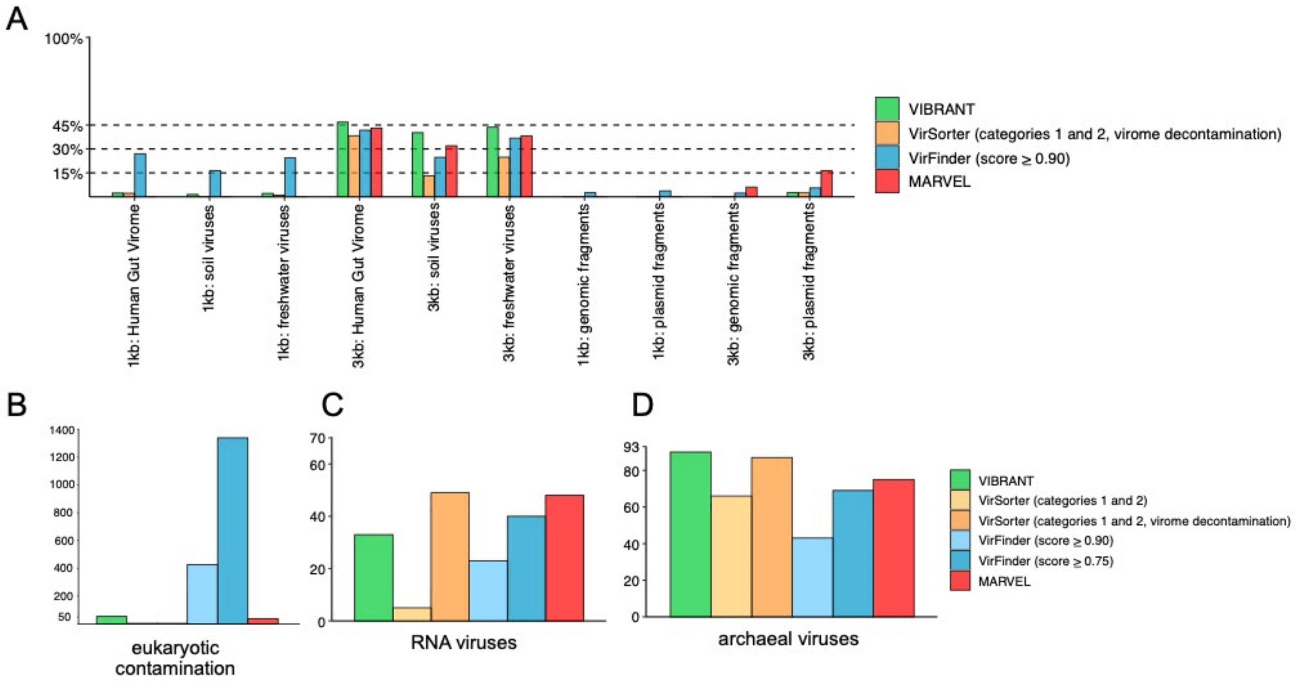

Figure S1. Comparison of VIBRANT, VirFinder, VirSorter and MARVEL on additional validation datasets. (A) The TPR and FPR of virus identifications for 1kb and 3kb scaffolds, (B) the effect of eukaryotic sequence contamination, and the ability to recover complete (C) RNA and (D) archaeal viruses.

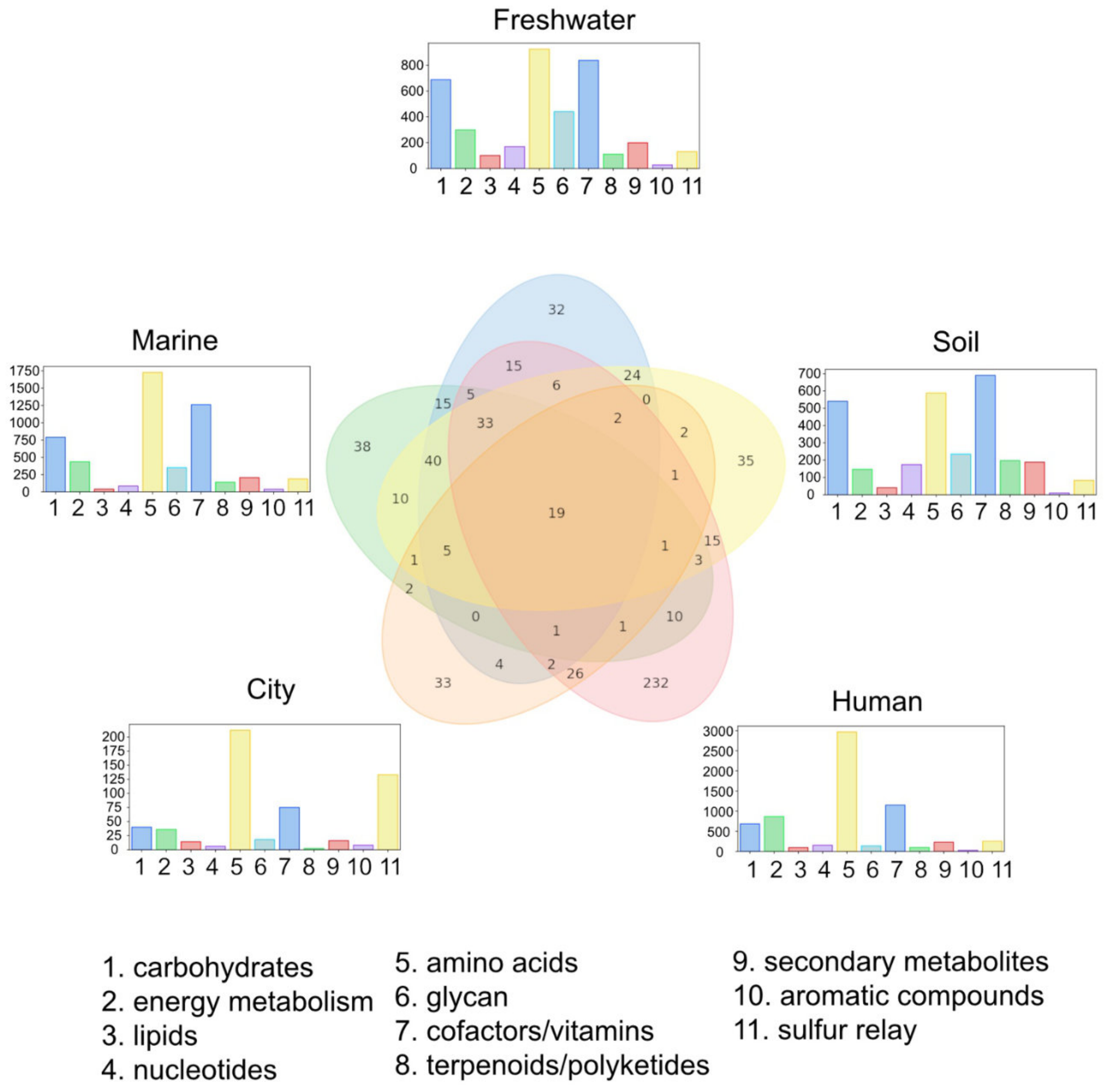

Figure S2. AMG and metabolic pathways between diverse environments. VIBRANT was used to predict viruses from IMG/VR datasets and the identified metabolic pathways and AMGs were compared for freshwater, marine, soil, city and human-associated environments (graphs). The respective AMGs and their abundances were likewise compared (venn diagram).

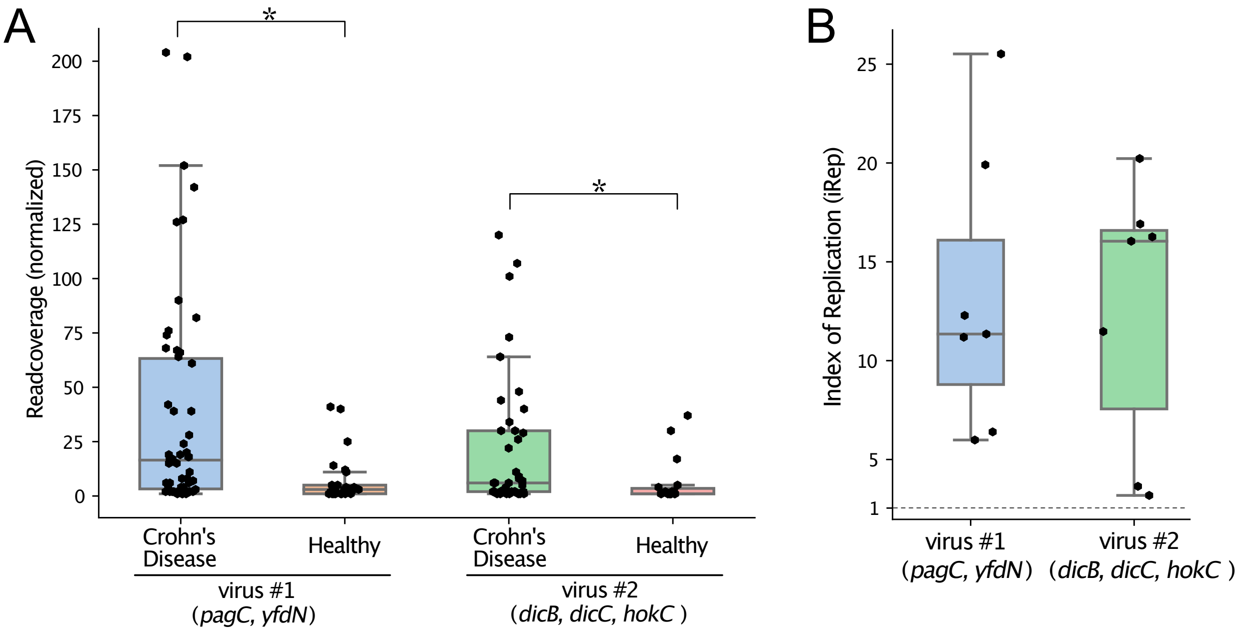

Figure S3. Differential abundance and activity of two viruses associated with Crohn's Disease. (A) Normalized read coverage of two abundant Crohn's-associated viruses that encode putative DAGs between Crohn's Disease and healthy gut metagenomes. Asterisks represent significant differential abundance ($p<0.05$). (B) iRep analysis for the same two viruses as (A), representative of seven metagenomes per virus for which the virus was in high abundance. The dotted line indicates an iRep value of one, or low to no activity (i.e., genome replication).

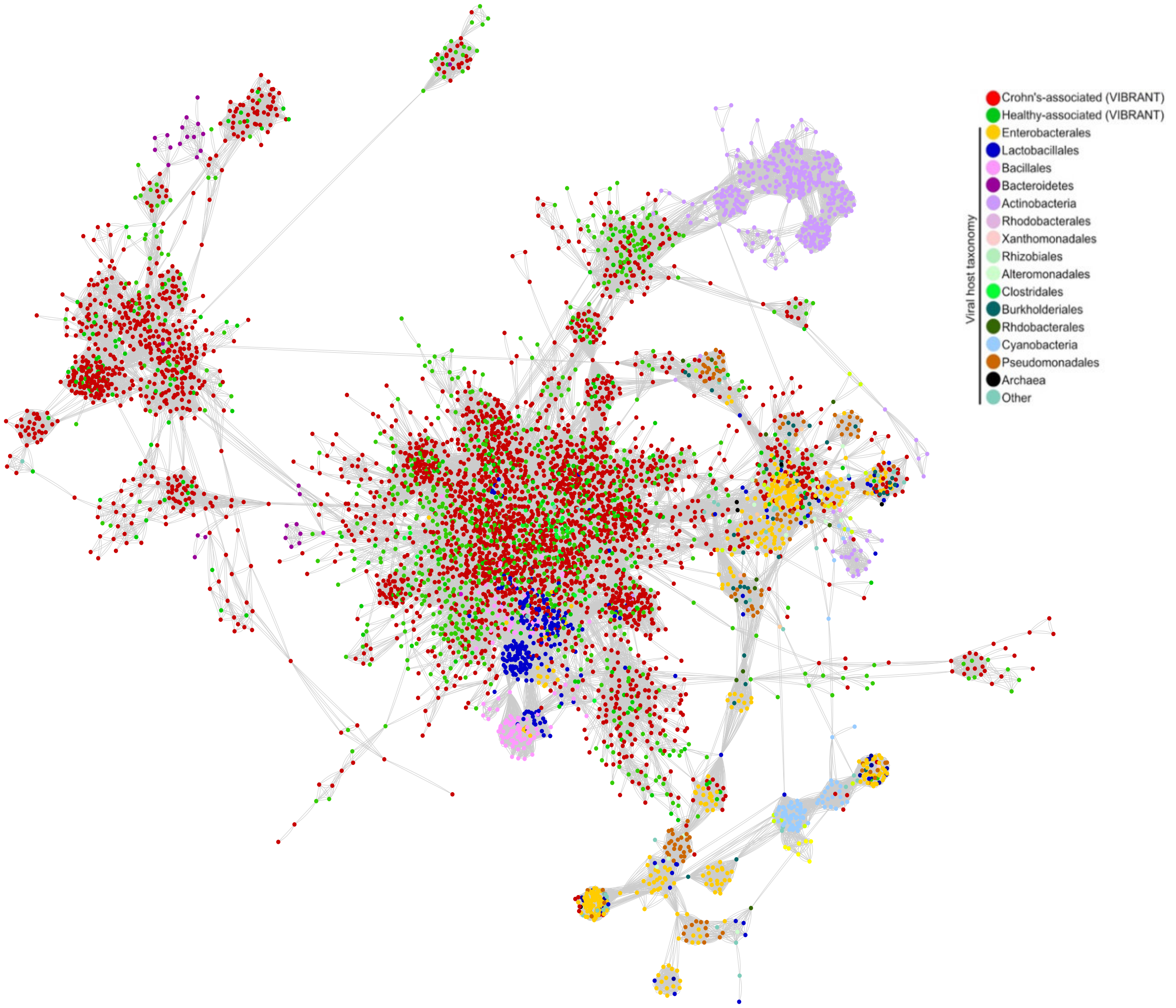

Figure S4. Protein network of two Crohn's Disease validation datasets. VIBRANT was used to predict viruses from two datasets for validation of marker virus and putative DAG discovery. The resulting viruses were used to construct a protein network indicating Crohn's-associated viruses clustering with enteroviruses more often than healthy-associated viruses.

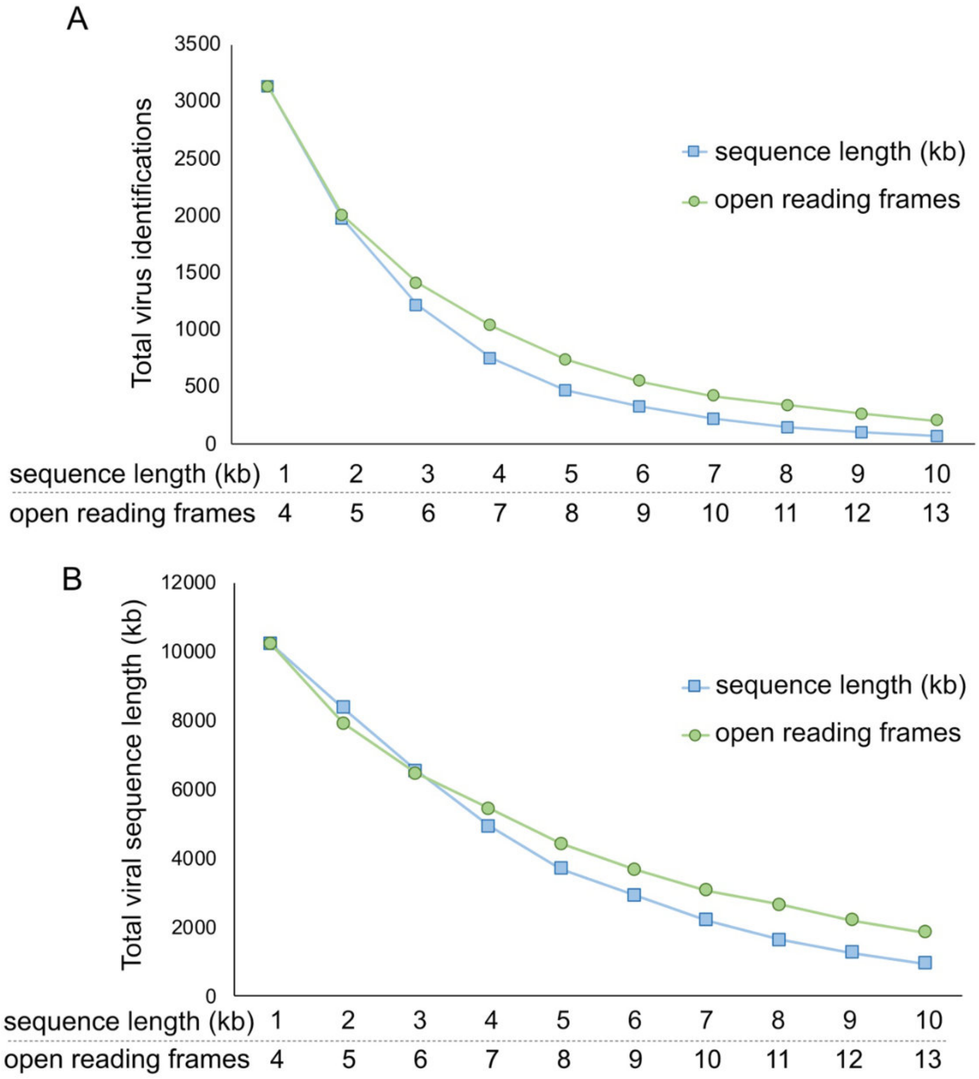

Figure S5. Comparison of limiting to sequence length or open reading frames. VIBRANT was used to predict viruses from an estuary virome and set to limit to either scaffold length or total encoded open reading frames. The (A) total virus identifications and (B) total viral sequence length were compared to show that limiting to open reading frames will typically yield more data.
